# Branched-chain amino acid catabolism promotes M2 macrophage polarization

**DOI:** 10.3389/fimmu.2024.1469163

**Published:** 2024-11-08

**Authors:** Manxi Lu, Da Luo, Zixuan Zhang, Feng Ouyang, Yihong Shi, Changyong Hu, Hang Su, Yining Li, Jiayi Zhang, Qian Gui, Tian-Shu Yang

**Affiliations:** ^1^ Shanghai Key Laboratory of Metabolic Remodeling and Health, Institute of Metabolism and Integrative Biology, Fudan University, Shanghai, China; ^2^ School of Life Sciences, Tianjian Laboratory of Advanced Biomedical Sciences, Zhengzhou University, Zhengzhou, Henan, China; ^3^ Shanghai Key Laboratory of Lung Inflammation and Injury, Department of Pulmonary Medicine, Zhongshan Hospital, Fudan University, Shanghai, China

**Keywords:** M2 macrophages, BCAA, SLC25A44, BCAT2, BCKDHA

## Abstract

**Introduction:**

During an immune response, macrophages undergo systematic metabolic rewiring tailored to support their functions. Branched-chain amino acid (BCAA) metabolism has been reported to modulate macrophage function; however, its role in macrophage alternative activation remain unclear. We aimed to investigate the role of BCAA metabolism in macrophage alternative activation.

**Method:**

The metabolomics of BMDM-derived M0 and M2 macrophages were analyzed using LC-MS. BCAAs were supplemented and genes involved in BCAA catabolism were inhibited during M2 macrophage polarization. The expression of M2 marker genes was assessed through RT-qPCR, immunofluorescence, and flow cytometry.

**Results and discussion:**

Metabolomic analysis identified increased BCAA metabolism as one of the most significantly rewired pathways upon alternative activation. M2 macrophages had significantly lower BCAA levels compared to controls. BCAA supplementation promoted M2 macrophage polarization both in vitro and in vivo and increased oxidative phosphorylation in M2 macrophages. Blocking BCAA entry into mitochondria by knockdown of SLC25A44 inhibited M2 macrophage polarization. Furthermore, M2 macrophages polarization was suppressed by knockdown of Branched-chain amino-acid transaminase 2 (BCAT2) and branched chain keto acid dehydrogenase E1 subunit alpha (BCKDHA), both of which are key enzymes involved in BCAA oxidation. Overall, our findings suggest that BCAA catabolism plays an important role in polarization toward M2 macrophages.

## 1 Introduction

Macrophages are crucial players during inflammatory processes with great functional plasticity. They initially reacts with a variety of antigens and dynamically change their functions over the course of an immune response, releasing cytokines and chemokines that activate other immune cells (1). During the inflammatory process, upon sensing classical activation signals associated with infection such as lipopolysaccharide (LPS, a bacterial cell wall component) and interferon-γ (IFN-γ), macrophages turn on pro-inflammatory functions to help eliminate pathogens and initiate adaptive immune responses (2). In contrast, in response to cytokines such as interleukin-4 (IL-4), macrophages are alternatively activated to reduce tissue damage and initiate tissue regeneration and remodeling (3). M2 macrophages are also important in the response to helminth and fungal infections (4, 5). An increasing amount of literature suggests that different functional states of macrophages are coupled with metabolic reprogramming, which plays an important role in orchestrating macrophage functions (6). For instance, one of the most distinctive metabolic characteristics between M1 and M2 macrophages is L-arginine metabolism. The catalytic enzymes responsible for L-arginine metabolism, inducible nitric oxide synthase (iNOS) and arginase 1, were recognized as hallmark effector molecules of M1 and M2 macrophages, respectively (7). Another example is the citrate cycle: while the citrate cycle is intact in M2 macrophages, M1 macrophages show disruptions at two points—after citrate and after succinate. These interruptions lead to the accumulation of metabolites such as citrate, itaconate and succinate, which support the inflammatory activation of M1 macrophages (8).

Branched-chain amino acid (BCAA), including leucine (leu), isoleucine (Ile) and valine (Val), are essential dietary amino acids participating in a variety of crucial biochemical functions (9). BCAAs are transported into mitochondria by solute carrier family 25, member 44 (SLC25A44) (10, 11). The first step of BCAA catabolism involves the conversion of BCAAs to branched-chain α-keto acids (BCKAs), including α-ketoisovalerate (KIV), α-keto-β-methylvalerate (KMV), α-ketoisocaproate (KIC), through transamination catalyzed by the branched-chain aminotransferases BCAT1 and BCAT2 in the cytoplasm and the mitochondria, respectively (12). BCKAs undergo further oxidative decarboxylation to form acyl-CoA derivatives and NADH through the branched-chain ɑ-ketoacid dehydrogenase (BCKDH) complex. This complex, situated in the mitochondria, serves as a rate-limiting enzyme in the oxidation of BCAAs (13).

Interestingly, several recent studies have provided evidence that BCAA metabolism may influence macrophage metabolism and activation. Exposure to BCKAs reduced the phagocytic activity of macrophages (14). Inhibition of BCAT1 activity led to the reduction of oxygen consumption, glycolysis and cell migration in macrophages *in vitro*, which was shown to be associated with reduced IRG1 levels and itaconate synthesis (15). High level of BCAAs (10 mM) stimulated the release of cytokines, such as IL-6, TNF-α, and ICAM-1, in peripheral blood mononuclear cells (PBMCs) (16). A recent study has shown that BCAA supplementation enhances both M1 and M2 polarization in macrophages during muscle repair. The mTORC1-HIF1α-glycolysis pathway is suggested to mediate the effects of BCAAs on M1 polarization, but not on M2 polarization (17). Thus, the mechanisms by which BCAAs, particularly BCAA catabolism, contribute to M2 polarization remains unclear.

In this study, we investigated the role of BCAA metabolism in M2 macrophage polarization. We found that the abundance of BCAAs was reduced in M2 macrophages compared to M0 macrophages. BCAA supplementation promoted M2 macrophage polarization both *in vitro* and *in vivo*. Furthermore, inhibition of BCAA catabolism by knockdown of SLC25A44, BCAT2 or BCKDHA suppressed M2 macrophage polarization, indicating that BCAA catabolism is important for M2 macrophage polarization. These data uncover an important role of BCAA catabolism in M2 macrophage polarization.

## 2 Materials and methods

### 2.1 Animals

6-8-weeks-old wild-type C57BL/6J mice were purchased from Jiangsu JicuiYaokang Biotechnology Co., Ltd (Jiangsu, China). The animals were kept in a pathogen-free environment. All experimental procedures were approved by the animal research committee of Fudan University. Both male and female mice are used for bone marrow isolation. Female mice are used for chitin administration experiments.

### 2.2 Cells

L-929 cells was obtained from the cell bank of the Chinese Academy of Science (Shanghai, China). Cells were cultured in RPMI 1640 medium (Gibco, USA) supplemented with 10% FBS (VivaCell, China) and 1% penicillin/streptomycin (Invitrogen, USA). Cells were maintained at 37°C in a humidified 5% CO_2_ atmosphere.

Bone marrow-derived macrophages (BMDMs) were obtained as previously described (18). Briefly, bone marrow cells were obtained from wild-type C57BL/6 mice aged 6 to 8 weeks and cultured in RPMI 1640 supplemented with 10% FBS, 1% penicillin/streptomycin and 30% supernatants of L-929 conditioned medium. On day 7, attached macrophages were washed and harvested for stimulation.

Peritoneal exudate cells (PECs) were isolated from peritoneum by lavage using 4 mL PBS for 3 times, and were collected for analysis.

### 2.3 BMDM polarization and treatment

Differentiated BMDMs were incubated for 24 hr in culture medium containing IL-4 (PeproTech, USA) at a concentration of 20 ng/mL to induce M2 macrophage polarization. BMDMs were treated by 100 ng/mL LPS and 50 ng/mL IFNγ to induce M1 macrophage polarization. In some experiments, BMDMs were cultured in BCAA free RPMI 1640 medium (USbiological, USA) with or without 0.2 mM, 0.4 mM, 0.8 mM Val, Leu, Ile (Sigma, USA) or their combination.

### 2.4 Gene knockdown assay using siRNA

BMDMs were transfected with 20 μM stock solution of small interfering RNAs (siRNA) for 24 hr by using Lipofectamine™ RNAiMAX Transfection Reagent (Invitrogen, USA) according to manufacturer’s instructions. Serum-free Opti-MEM (Gibco, USA) was used to dilute the siRNA reagents. si-SLC25A44, si-BCAT2, si-BCKDHA and si-NC oligo were purchased from GenePharma (Shanghai, USA). The mouse SLC25A44 siRNA sequence was 5′-CUCUCGGCAAGAAUCAUCUTT-3′, The mouse BCAT2 siRNA sequence was 5′-GGGAGAACCUUGGCUUCUUCU-3′, The mouse BCDKHA siRNA sequence was 5′-GCUΜGAGUUCAUCCAGCCCAA-3′. After 24 hr, the cells were cultured with IL-4 (20 ng/mL) to induce their polarization into M2.

### 2.5 Real-time quantitative PCR

Total RNA was extracted from BMDMs using TRIzol reagent (Invitrogen, USA) according to the manufacturer’s instructions. cDNA was synthesized with PrimeScript RT Master Mix (TaKaRa, Japan). Quantitative RT-PCR was performed using SYBR Green Master Mix (Yeasen, China). The relative mRNA expression level was calculated by the 2^-ΔΔCt^ method (fold expression), *β-actin* was used as an internal reference. The sequences of primers are listed in **Supplementary Table 1**.

### 2.6 Immunofluorescence

For immunofluorescence, BMDMs were seeded in coverslips. After adhesion, cells were polarized and treated with BCAAs. Cells were then fixed using 4% PFA for 20 min, rinsed three times with PBS, and permeabilized and blocked with PBS containing 1%BSA and 0.3%Triton X-100 for 1 hr at room temperature. The cells were then stained using a rabbit polyclonal anti-RELMα antibody (Peprotech, USA) at 1:200 dilution in a humid chamber at 4°C overnight.

After primary antibody incubation, macrophages were stained with rabbit anti-IgG (Alexa Fluor^®^ 488, Invitrogen, USA) at 1:200 dilution at room temperature in the dark for 1 hr. The nuclear staining was done using Mounting Medium containing DAPI (Yeasen, China). Images were assessed by fluorescence microscopy with a ×40 objective lens (EVOS™ M7000, Thermo, USA). Quantification of the images was performed using Image J software.

### 2.7 Flow cytometry

Antibodies for flow cytometry were purchased from Thermo Fisher Scientific. The antibodies used were anti-CD45 (PE), anti-F4/80 (FITC), anti-Siglec-F (PE-Cyanine7), anti-CD11b (APC- Cyanine7), anti-CD206 (APC). Cells were washed in FACS buffer (3% (vol/vol) FBS and 1 mM EDTA in PBS, pH 7.4) once, then incubated with each antibody in 50 μL FACS buffer for 30 min and washed once with 1 mL FACS buffer. Cells were analyzed on the BD LSRII flow cytometer, and data analyzed using Flowjo software.

### 2.8 Chitin administration

10 mg chitin (Solarbio, China) was suspended in 3 mL PBS, sonicated with a JY92-IIDN device (SCIENTZ, China) at 150 W, work 7 s and pause 6 s two times for 10 min. After filtration with 100 μm cell strainer, chitin was diluted in 10 mL PBS, 100 μL of chitin was injected once intraperitoneally and PECs were collected 2 d after administration (19, 20).

### 2.9 BCAA supplementation

BCAA (valine, leucine, isoleucine) solution was prepared in PBS (pH 7.4) as isomolar mixtures and sterilized by filtration through a 0.22 μm filter. Mice were intraperitoneally injected with 500 μL of 30 μmol, 60 μmol or 90 μmol BCAA solution (21) after chitin administration.

### 2.10 Metabolomics

IL-4 treated BMDMs or non-treated BMDMs were washed once with PBS. Pre-chilled 80% (vol/vol) methanol was added and then incubated at -80°C overnight. The metabolite-containing supernatants were centrifuged for 10 min at 14000 g and 4°C, and supernatants were dried under nitrogen flow (22). The metabolome extracts were reconstituted in acetonitrile and H_2_O (1:1, vol/vol), vortex for 1 min, and centrifuged for 15 min at 14000 rpm and 4°C. Targeted LC-MS were performed by QTRAP 6500+ Liquid chromatography-mass spectrometer (AB SCIEX, USA) interfaced with ExionLC™ AD HPLC system (AB SCIEX, USA). Mobile phases A (95% H2O; 5% acetonitrile; 20 mM ammonium acetate; 10 mM ammonia; 50 µL 0.05 M medronic acid) and mobile phases B (100% acetonitrile) are used. Chromatographic separation was performed on a HILIC column (iHILIC-(P) Classic column, 5 μm, 150 × 2.1 mm) with the following gradient: 0-2 min 15% A; 2-7 min 15-40% A; 7-12 min 40-65% A; 12-12.1 min 65-80% A; 12.1-15.9 min 80% A; 15.9-16 min 80-15% A; 16-23 min 15% A. The flow rate was set as 0.2 mL/min, and the injection volume was 5 µL. The column temperature was 30°C and autosampler temperature was 6°C. The missing value was addressed according to the 80% rule (23): a metabolite was considered detectable if it appeared in at least 4 out of 5 samples in a group. In order to remove potential variations in total metabolite abundance across samples, the total intensity of metabolite peaks within each sample was adjusted to the mean total intensity across all samples. Each metabolite peak intensity is then divided by the normalization factor, standardizing the total metabolite abundance within each sample (24). The peak area list with compound names was analyzed by MetaboAnalyst 6.0. Kyoto Encyclopedia of Genes and Genomes (KEGG) was used for the enrichment analysis of the significantly upregulated or downregulated metabolites.

### 2.11 Oxygen consumption assay

The oxygen consumption rate (OCR) was measured by the Seahorse XFe96 Extracellular Flux Analyzer (Agilent, USA). BMDM cells were plated at 8 × 10^4^ cells/well in a Seahorse 96-well microplate (Agilent, USA) and treated as indicated. The cells were incubated in Seahorse XF DMEM medium (Agilent, USA) for 1 hr at 37°C in measuring chamber before the assay. Oxygen consumption rate was measured under basal conditions and in response to sequential injections of 1 μM oligomycin, 1.2 μM FCCP (Carbonyl cyanide 4-trifluoromethoxy phenylhydrazone), and 1 μM rotenone.

### 2.12 Data analyses and statistics

Statistical analysis was performed with GraphPad Prism 9 software. Data are shown as mean ± SEM. Groups were compared by a Student’s two-tailed *t* test and two way ANOVA (followed by Tukey’s multiple comparison tests). *P* values of < 0.05 were considered to be statistically significant. The number of experiments and significance levels are presented in the legend of the figures.

## 3 Results

### 3.1 Decreased BCAAs in M2 polarized macrophages

**Figure 1:**
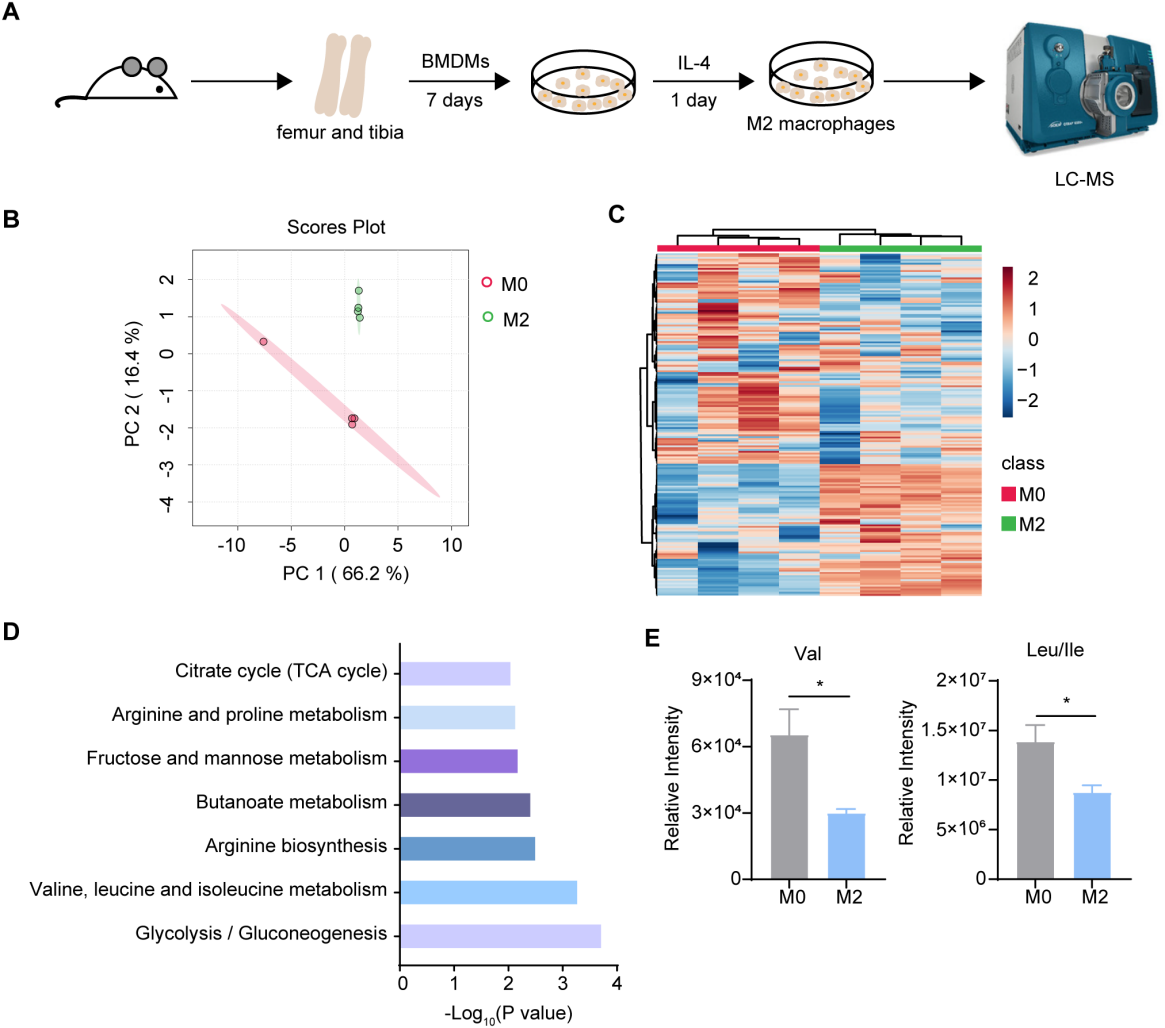
Metabolomics analysis between M0 and M2 BMDM. **(A)** The schematic protocol of BMDM differentiation and M2 polarization in 6-8 week-old female mice (n = 4 replicates). **(B)** Principal Component Analysis (PCA) of M0 and M2 BMDM metabolites (n = 4 replicates). **(C)** Heatmap of the intracellular metabolites in M0 and M2 BMDM. **(D)** KEGG pathway analysis of changed differential metabolites (*P* < 0.05) (n = 4 replicates). **(E)** Val, Leu and Ile relative intensity in M0 and M2 BMDM (n = 4 replicates). Data are shown as mean ± SEM. Statistics were performed using two-tailed Student’s *t* test. *P < 0.05.

To systematically identify the metabolic pathways that are rewired in macrophages upon alternative activation, we performed metabolomic analysis comparing murine bone marrow-derived macrophages (BMDMs) stimulated with IL-4 for 24 hr (M2) to unstimulated BMDMs (M0) (**Figure 1A**). We totally detected 186 metabolites in the samples, and we identified upregulated or downregulated metabolites in M2 relative to M0 cells (>1.5-fold, *P* < 0.05). Principal component analysis (PCA) and heatmap of the metabolites abundance revealed a significant separation between M0 and M2 macrophages (**Figures 1B, C**). In addition to several pathways including citrate cycle and arginine metabolic pathway, which are known to be altered in M2 macrophages, we discovered that BCAA metabolism was one of the most profoundly impacted pathways (**Figure 1D**). The abundance of Val and Leu/Ile were significantly decreased in M2 macrophages compared to M0 macrophages (**Figure 1E**). Additionally, one of the M0 samples did not cluster with the others in the PCA plot (**Figure 1B**). To determine if this outlier affected BCAA levels, we analyzed the relative intensities of Val and Leu/Ile both with and without this M0 outlier. The results showed a significant decrease in M2 macrophages, regardless of whether the outlier was included (**Supplementary Figure 1**). These results suggested that BCAA levels were decreased in M2 macrophages.

### 3.2 BCAA supplementation promoted M2 macrophage polarization both *in vivo* and *in vitro*

**Figure 2:**
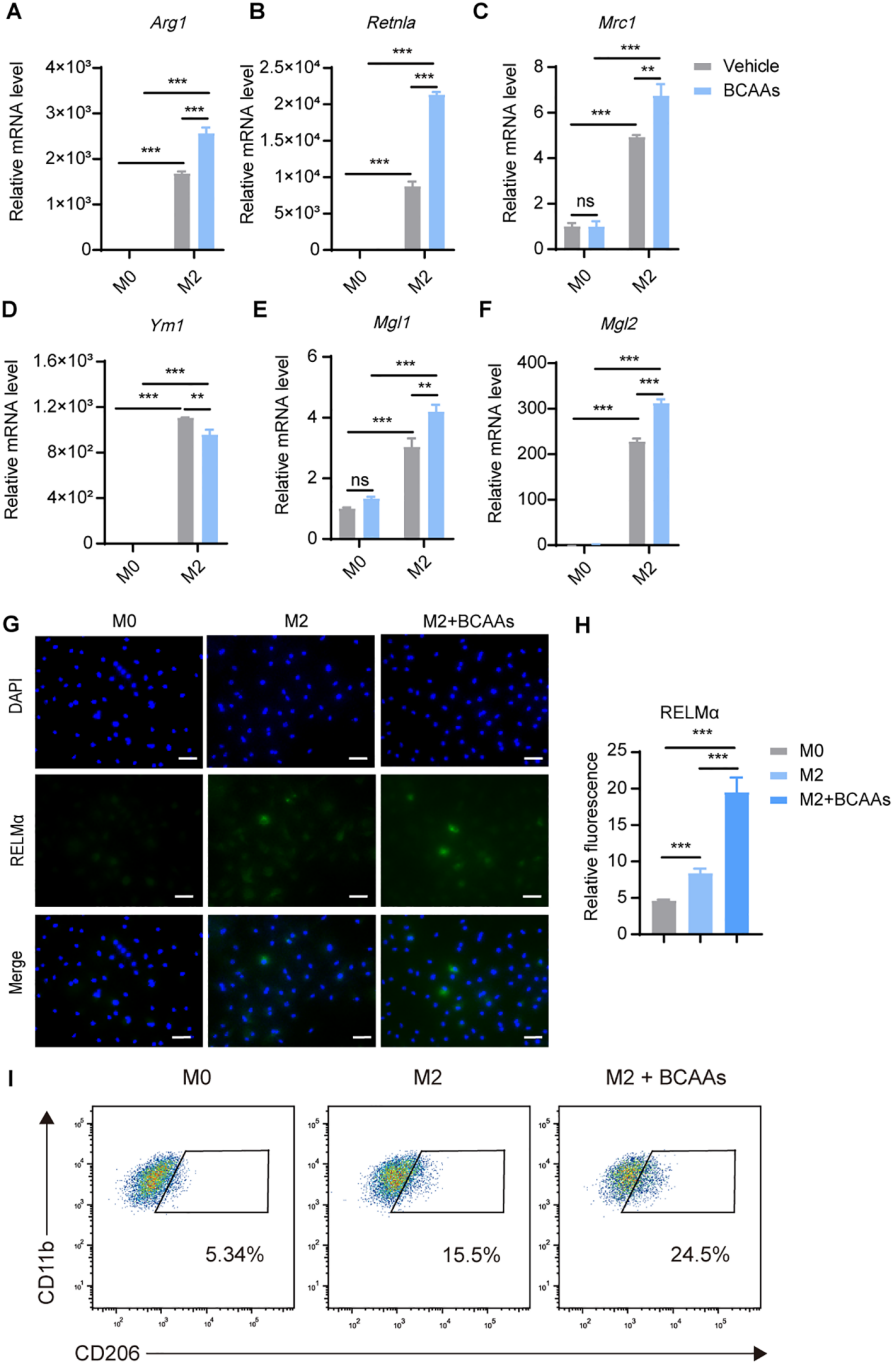
BCAAs enhance M2 polarization. BMDMs were treated with 0.8 mM BCAAs under M0 or M2 conditions for 24 hr. mRNA expression levels of *Arg1* **(A)**, *Retnla* **(B)**, *Mrc1* **(C)**, *Ym1* **(D)**, *Mgl1* **(E)** and *Mgl2* **(F)** were assessed by RT-qPCR (n = 3 replicates). **(G)** BMDMs were treated with IL-4 (20 ng/mL) in the presence or absence of BCAAs (0.8 mM) for 24 hr and subjected to immunofluorescence using anti-RELMα antibody (green) and DAPI (blue). Scale bar, 20 μm. **(H)** Quantification of relative fluorescent signal for RELMα in BMDMs from the M0, M2 and M2 + BCAAs groups (n = 5 replicates). **(I)** Flow cytometric analysis of CD11b^+^F4/80^+^CD206 ^+^cells were shown. Data are shown as mean ± SEM. Statistics were performed using two way ANOVA followed by Tukey multiple comparisons. **P < 0.01; ***P < 0.001; ns, not significant. *Arg1*, Arginase 1; *Retnla*, Resistin like alpha; *Mrc1*, Mannose receptor, C type 1; *Ym1*, Chitinase 3 like 1; *Mgl1*, Macrophage galactose N-acetyl-galactosamine specific lectin 1; *Mgl2*, Macrophage galactose N-acetyl-galactosamine specific lectin 2.

We next determined whether BCAAs affect M2 macrophage polarization. Differentiated BMDMs were treated with 20 ng/mL of IL-4 for 24 hr with or without BCAAs. The mRNA expression level of the key M2 macrophage genes were determined by RT-qPCR. Treatment with BCAAs increased the mRNA expression levels of *Arg1*, *Retnla*, *Mrc1*, *Mgl1* and *Mgl2* in M2 macrophages, but did not increase the expression level of *Ym1*. BCAAs did not change the expression of M2 macrophage-related genes under M0 conditions (**Figures 2A–F**). In addition, BCAAs treatment increased RELMα protein level as assessed by immunofluorescence (**Figures 2G, H**), as well as the frequency of CD206^+^CD11b^+^F4/80^+^ cells (**Figure 2I**). The gating strategy was shown in **Supplementary Figure 2**.

**Figure 3:**
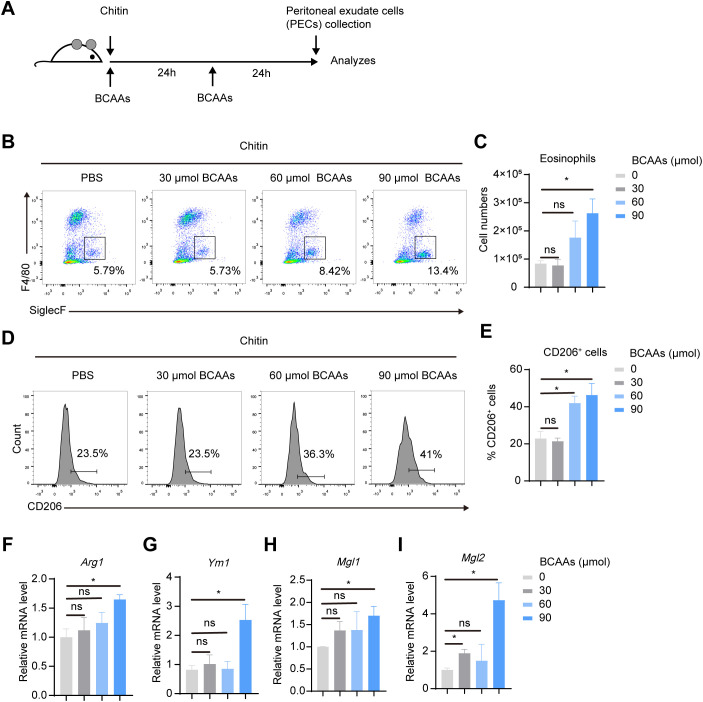
BCAAs promote M2 macrophage polarization in chitin-treated mice in a dose-dependent manner. **(A)** Schematic diagram of the experimental protocol. Mice were intraperitoneally injected with 100 μL chitin. 30, 60 and 90 μmol BCAAs were administrated at 0 and 24 hr after chitin administration. PECs were collected and analyzed at 24 and 48 hr post chitin administration. **(B)** Eosinophil populations in the peritoneal cavity were analyzed by flow cytometry. Representative flow plots of gated CD45^+^ cells were shown. **(C)** Total numbers of eosinophils (n = 4 independent biological replicates). **(D–E)** Flow cytometry analysis **(D)** and percentage **(E)** of CD206^+^ cells in gated CD11b^+^ F4/80^+^ PECs (n = 4 independent biological replicates). **(F–I)** mRNA expression levels of *Arg1* **(F)**, *Ym1* **(I)**, *Mgl1* **(H)** and *Mgl2* **(I)** in PECs was determined by quantitative PCR (n = 4 independent biological replicates). Data are shown as mean ± SEM. Statistics were performed using two-tailed Student’s *t* test. *P < 0.05; *Arg1*, Arginase 1; *Ym1*, Chitinase 3 like 1; *Mgl1*, Macrophage galactose N-acetyl-galactosamine specific lectin 1; *Mgl2*, Macrophage galactose N-acetyl-galactosamine specific lectin 2.

To understand the dose-dependent effect of BCAAs on M2 macrophage polarization, we treated BMDMs with increasing doses of BCAAs for 12, 24 and 48 hours during M2 macrophage polarization. The result showed that BCAAs increased *Retnla* expression in a dose-dependent manner at both 24 and 48 hours and increased *Arg1* expression at 24 hours. In contrast, BCAA treatment suppressed *Ym1* expression at 24 and 48 hours. At the 12 hour-time point, BCAA treatment had no significant effect on the expression of *Arg1*, *Retnla* or *Ym1* (**Supplementary Figure 3**). In addition, to understand whether BCAA supplementation specifically modulate M2 macrophage polarization, we also determined the effect of BCAA supplementation on M1 macrophage polarization. We found that BCAA supplementation didn’t increase the expression level of M1 markers in polarized M1 macrophages, including *Il1β*, *Il6*, *Nos2*, *Tnfa* (**Supplementary Figure 4**). Next, to investigate the functional role of BCAAs in alternatively activated macrophages polarization *in vivo*, we subjected wildtype (WT) mice to an *in vivo* model of chitin-induced M2 polarization. Chitin, a major structural component of helminths and fungi, was suggested to induce M2 macrophage polarization and subsequent eosinophil recruitment when injected peritoneally (19, 20). We administered mice with chitin along with increasing doses of BCAAs (**Figure 3A**). The results indicate that BCAAs start to promote M2 macrophage polarization in peritoneal macrophages at a dose of 90 µmol, corresponding to 4.5 mM in circulation, given that the average mouse body weight is approximately 20 g. We found that chitin-induced recruitment of eosinophils was significantly increased in BCAAs treated animals (**Figures 3B, C**). BCAA supplementation increased the percentage of CD11b^+^F4/80^+^CD206^+^ cells in PECs (**Figures 3D, E**). In addition, the expression of M2 macrophage-associated genes *Arg1*, *Ym1*, *Mgl1* and *Mgl2* in chitin-elicited PECs were also increased in mice administrated with BCAAs (**Figures 3F–I**). Additionally, we measured *Ym1* expression in peritoneal cells at 24 and 48 hours after chitin administration in mice treated with 90 µmol BCAAs. The results showed that *Ym1* levels decreased at 24 hours in mice treated with both chitin and BCAAs, but increased at 48 hours compared to mice treated with chitin alone. This result, at least in part, suggests that the varying effects of BCAA supplementation on *Ym1* expression may be influenced by the timing of detection (**Supplementary Figure 5**). Collectively, these data indicated that BCAAs promoted M2 macrophage *in vitro* and *in vivo*.

### 3.3 Leu promoted M2 macrophage polarization most robustly among BCAAs

**Figure 4:**
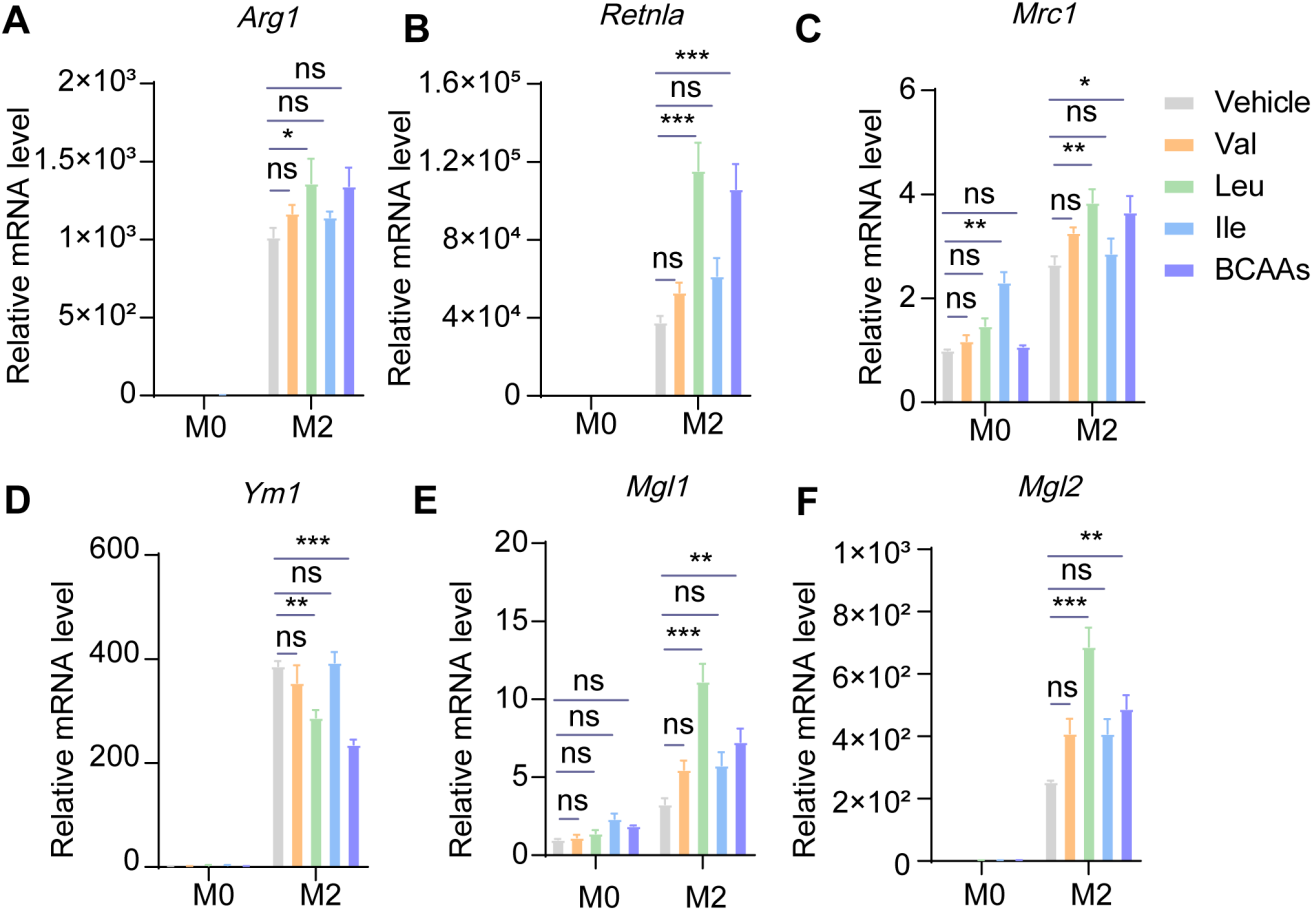
Leucine may dominate the effect of promoting M2 polarization among three kinds of BCAAs. BMDMs were treated with or without 0.8 mM of Val, Leu, Ile or their mixture under M0 or M2 conditions for 24 hr. The mRNA expression levels of *Arg1* **(A)**, *Retnla* **(B)**, *Mrc1* **(C)**, *Ym1* **(D)**, *Mgl1* **(E)** and *Mgl2* **(F)** were assessed by RT-qPCR (n = 3 replicates). Data are shown as mean ± SEM. Statistics were performed using two way ANOVA followed by Tukey multiple comparisons. *P < 0.05; **P < 0.01; ***P < 0.001; ns, not significant. *Arg1*, Arginase 1; *Retnla*, Resistin like alpha; *Mrc1*, Mannose receptor, C type 1; *Ym1*, Chitinase 3 like 1; *Mgl1*, Macrophage galactose N-acetyl-galactosamine specific lectin 1; *Mgl2*, Macrophage galactose N-acetyl-galactosamine specific lectin 2.

To explore the impact of individual BCAAs on M2 macrophage polarization, we supplemented BMDMs with leu, Ile and Val, either individually or in combination, under M0 or M2 conditions. We found that while Ile and Val exhibited some capacity to enhance M2 macrophage polarization, their effects were not as pronounced as those of Leu alone or the combination of all three BCAAs. The results suggested that Leu exerts the most pronounced effect on alternative macrophage polarization (**Figure 4**).

### 3.4 BCAA supplementation promoted oxidative phosphorylation in M2 macrophages

**Figure 5:**
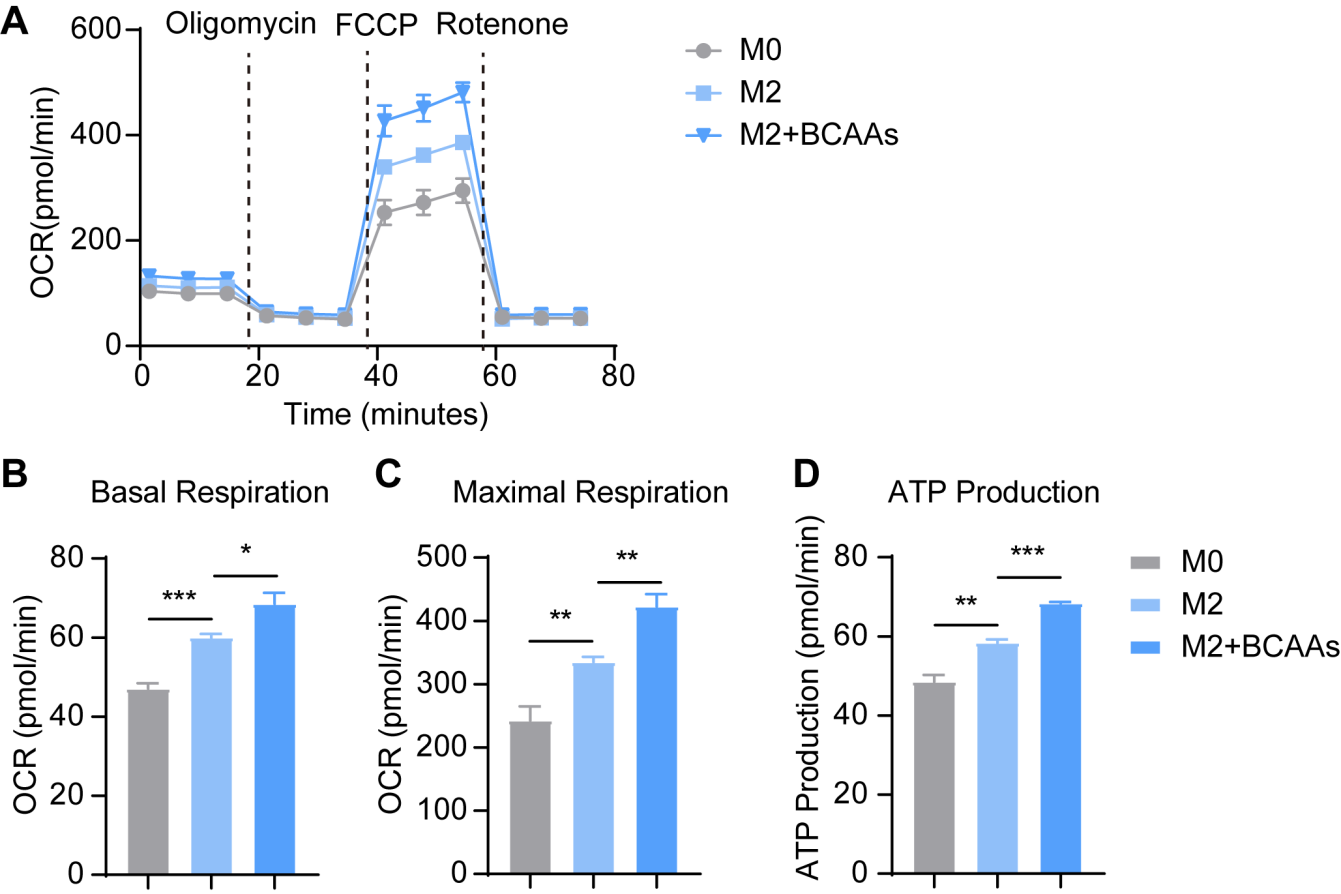
BCAAs increase OCR of M2 BMDM. BMDMs were treated with 0.8 mM BCAAs under M2 conditions for 24 hr. **(A)** The effect of BCAA supplementation on OCR of M2 BMDM (n = 4 replicates); The effect of BCAA supplementation on basal respiration **(B)**, maximal respiration **(C)** and ATP production **(D)** of M2 BMDM (n = 4 replicates). Data are shown as mean ± SEM. Statistics were performed using two-tailed Student’s *t* test. *P < 0.05; **P < 0.01; ***P < 0.001.

The metabolites of BCAA catabolism, such as acetyl-CoA and succinyl-CoA, enter the TCA cycle and fuel oxidative phosphorylation (OXPHOS), which is established to be critical for M2 polarization (25). Notably, the relative level of acetyl-CoA was upregulated in M2 macrophages, indicating enhanced OXPHOS in M2 macrophages (**Supplementary Figure 6**). We speculated BCAA catabolism serves to fuel the OXPHOS and supports M2 polarization. Thus, we tested the oxygen consumption rate (OCR) by Seahorse XFe96 Analyzer. OCR of M2 BMDMs increased strongly, and BCAAs enhanced the OCR of M2 BMDMs (**Figures 5A–D**), basal respiration, maximal respiration and ATP production all upregulated. Hence, BCAA supplementation promoted oxidative phosphorylation in M2 macrophages.

### 3.5 M2 polarization influenced BCAA metabolic gene expression

SLC25A44 transports BCAAs into mitochondria (10). BCAAs are transaminated by BCATs to form BCKAs, which is reversible. BCAT2 plays the role in mitochondria, while BCAT1 works in cytoplasm. BCKDH catalyzes the irreversible oxidation of BCKAs. The metabolite of BCAA catabolism enter TCA cycle as acetyl-CoA and succinyl-CoA. As BCAAs were reduced in M2 BMDMs (**Figure 1**) and supplementation of BCAAs promoted M2 polarization (**Figure 2**), we further examined the expression level of key genes in BCAA catabolism, including *Bcat1*, *Bcat2*, *Bckdha* and *Slc25a44* at 0, 4, 8, and 24 hours after IL-4 treatment. The result showed that *Bcat1* was nearly undetectable in BMDMs, while *Bcat2*, *Bckdha* and *Slc25a44* were all expressed (**Supplementary Figure 7A**). The mRNA levels of *Bcat1* and *Slc25a44* increased after 4 hours of IL-4 stimulation and then decreased over time. In contrast, *Bcat2* mRNA levels decreased following IL-4 treatment, while *Bckdha* levels remained unchanged until 24 hours, when they also decreased (**Supplementary Figures 7B–E**). We have assessed the protein level of BCKDHA in M0 and IL-4 induced M2 macrophages, and the protein level of BCKDHA was upregulated from 2 to 8 hours after IL-4 treatment and returned to baseline levels 24 hours post IL-4 treatment (**Supplementary Figure 8**). These results suggest that the expression level of key enzymes and mitochondrial transporter involved in BCAA catabolism were unchanged or moderately downregulated.

### 3.6 Inhibition of BCAA transportation mediated by SLC25A44 reduced M2 macrophage polarization

**Figure 6:**
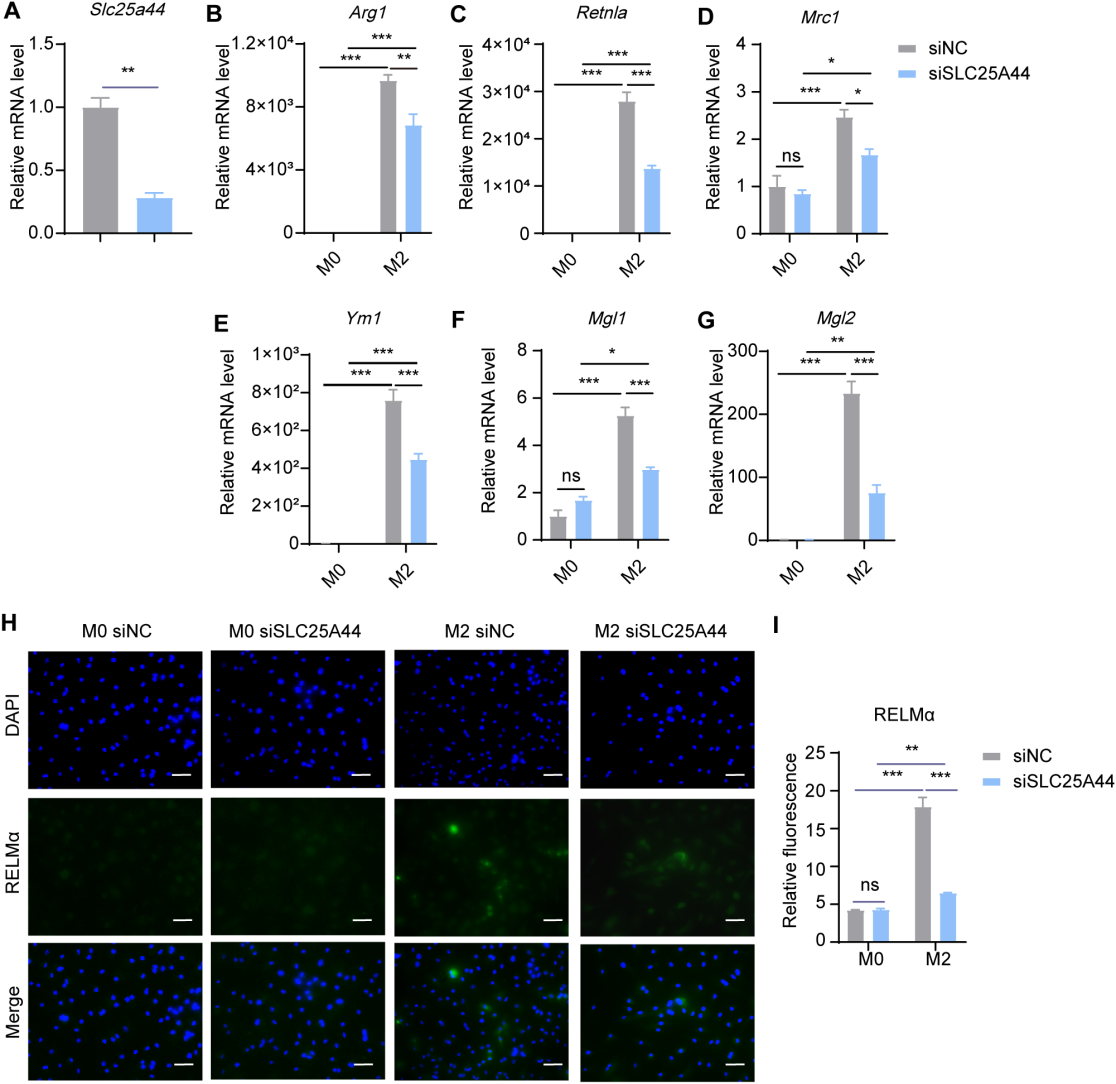
*Slc25a44* knockdown inhibits M2 polarization. BMDMs were transfected with siRNA targeting *Slc25a44* (siSLC25A44) and scrambled control siRNA (siNC) followed by M2 polarization. The mRNA expression levels of *Slc25a44* **(A)**, *Arg1* **(B)**, *Retnla* **(C)**, *Mrc1* **(D)**, *Ym1* **(E)**, *Mgl1* **(F)** and *Mgl2* **(G)** were assessed by RT-qPCR (n = 3 replicates). **(H)** BMDMs were treated with IL-4 (20 ng/mL) or not for 24 hr and subjected to immunofluorescence using anti-RELMα antibody (green) and DAPI (blue). Scale bar, 20 μm. **(I)** Quantification of relative fluorescent signal for RELMα in BMDMs from siNC and siSLC25A44 groups (n = 5 replicates). Data are shown as mean ± SEM. Statistics were performed using two way ANOVA followed by Tukey multiple comparisons. *P < 0.05; **P < 0.01; ***P < 0.001; ns, not significant. *Arg1*, Arginase 1; *Retnla*, Resistin like alpha; *Mrc1*, Mannose receptor, C type 1; *Ym1*, Chitinase 3 like 1; *Mgl1*, Macrophage galactose N-acetyl-galactosamine specific lectin 1; *Mgl2*, Macrophage galactose N-acetyl-galactosamine specific lectin 2.

To explore the effect of BCAA transport into mitochondria on M2 macrophage polarization, we knocked down *Slc25a44* with siRNA in BMDMs, and then polarized BMDMs toward M2 condition with 20 ng/mL IL-4 for 24 hr post transfection. With approximately 60% RNA silence efficiency, *Slc25a44* inhibition reduced the mRNA expression level of *Arg1*, *Retnla*, *Mrc1*, *Ym1*, *Mgl1* and *Mgl2* (**Figures 6A–G**). Additionally, knockdown of *Slc25a44* decreased the protein level of RELMα in M2 macrophages, as assessed by immunostaining (**Figures 6H, I**). The results suggested that inhibiting BCAA transport into mitochondria via SLC25A44 impairs BMDM M2 polarization.

### 3.7 Restraining BCAA transamination downregulated M2 macrophage polarization

**Figure 7:**
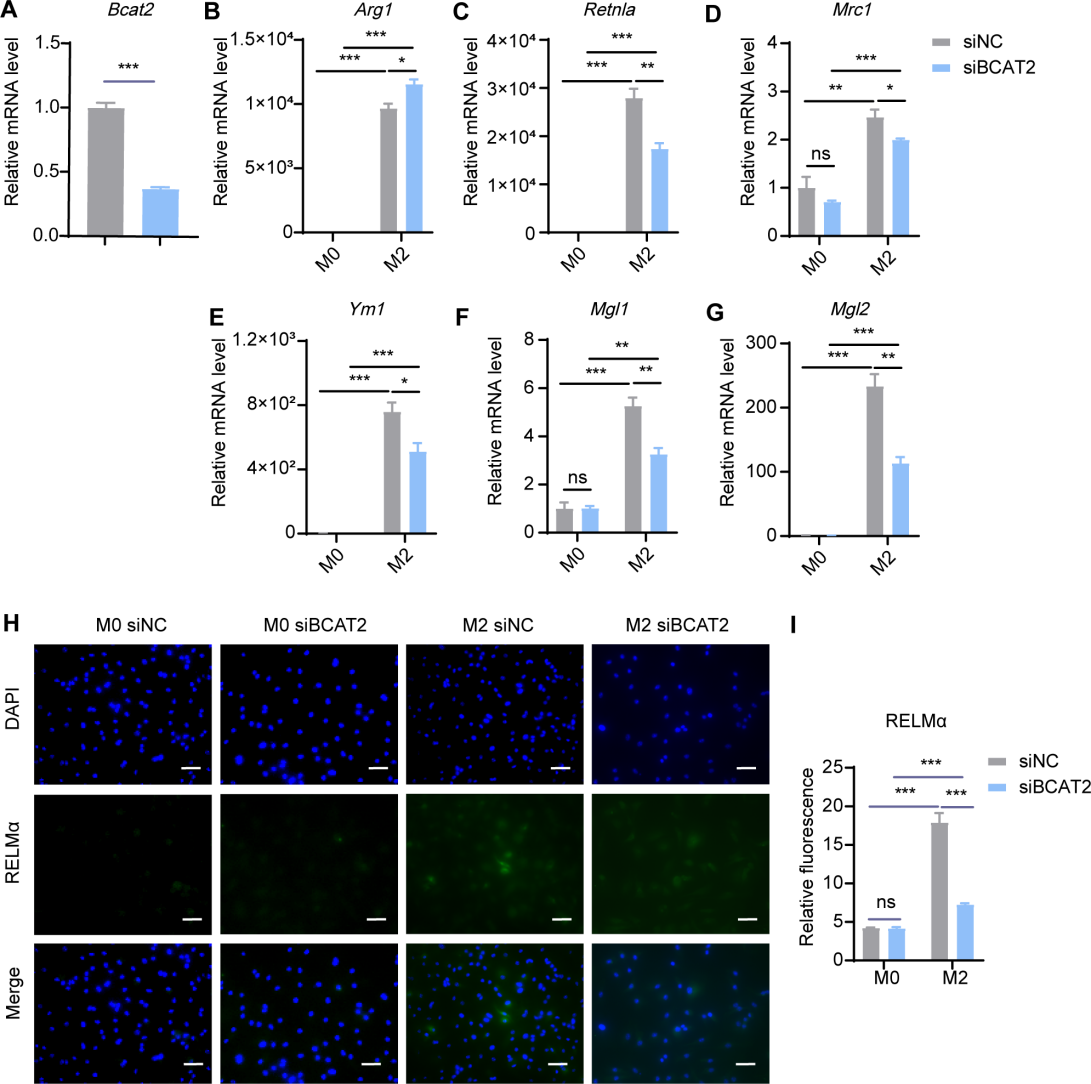
*Bcat2* knockdown inhibits M2 polarization. BMDMs were transfected with siRNA targeting *Bcat2* (siBCAT2) and scrambled control siRNA (siNC) followed by M2 polarization. The mRNA expression levels of *Bcat2* **(A)**, *Arg1* **(B)**, *Retnla* **(C)**, *Mrc1* **(D)**, *Ym1* **(E)**, *Mgl1* **(F)** and *Mgl2* **(G)** were assessed by RT-qPCR (n = 3 replicates). **(H)** BMDMs were treated with IL-4 (20 ng/mL) or not for 24 hr and subjected to immunofluorescence using anti-RELMα antibody (green) and DAPI (blue). Scale bar, 20 μm. **(I)** Quantification of relative fluorescent signal for RELMα in BMDMs from siNC and siBCAT2 groups (n = 5 replicates). Data are shown as mean ± SEM. Statistics were performed using two way ANOVA followed by Tukey multiple comparisons. **P < 0.01; ***P < 0.001; ns, not significant. *Arg1*, Arginase 1; *Retnla*, Resistin like alpha; *Mrc1*, Mannose receptor, C type 1; *Ym1*, Chitinase 3 like 1; *Mgl1*, Macrophage galactose N-acetyl-galactosamine specific lectin 1; *Mgl2*, Macrophage galactose N-acetyl-galactosamine specific lectin 2.

In mitochondria, BCAAs were transaminated by BCAT2 to generate BCKAs. To investigate the effect of BCAA transamination on M2 macrophage polarization, we knocked down *Bcat2* with siRNA and subsequently induced M2 polarization in BMDMs. We found that siRNA-mediated inhibition of BCAT2 resulted in a significant decrease in the expression levels of M2 marker genes, including *Retnla*, *Ym1*, *Mgl1* and *Mgl2*, but there were no obvious decrease in *Arg1* and *Mrc1*, (**Figures 7A–G**). Meanwhile, the protein levels of RELMα were reduced in M2 macrophages following *Bcat2* knockdown (**Figures 7H, I**). These results indicated that restraining BCAA transamination via BCAT2 reduced M2 macrophage polarization.

### 3.8 Suppressing BCAA decarboxylation dampened M2 macrophage polarization

**Figure 8:**
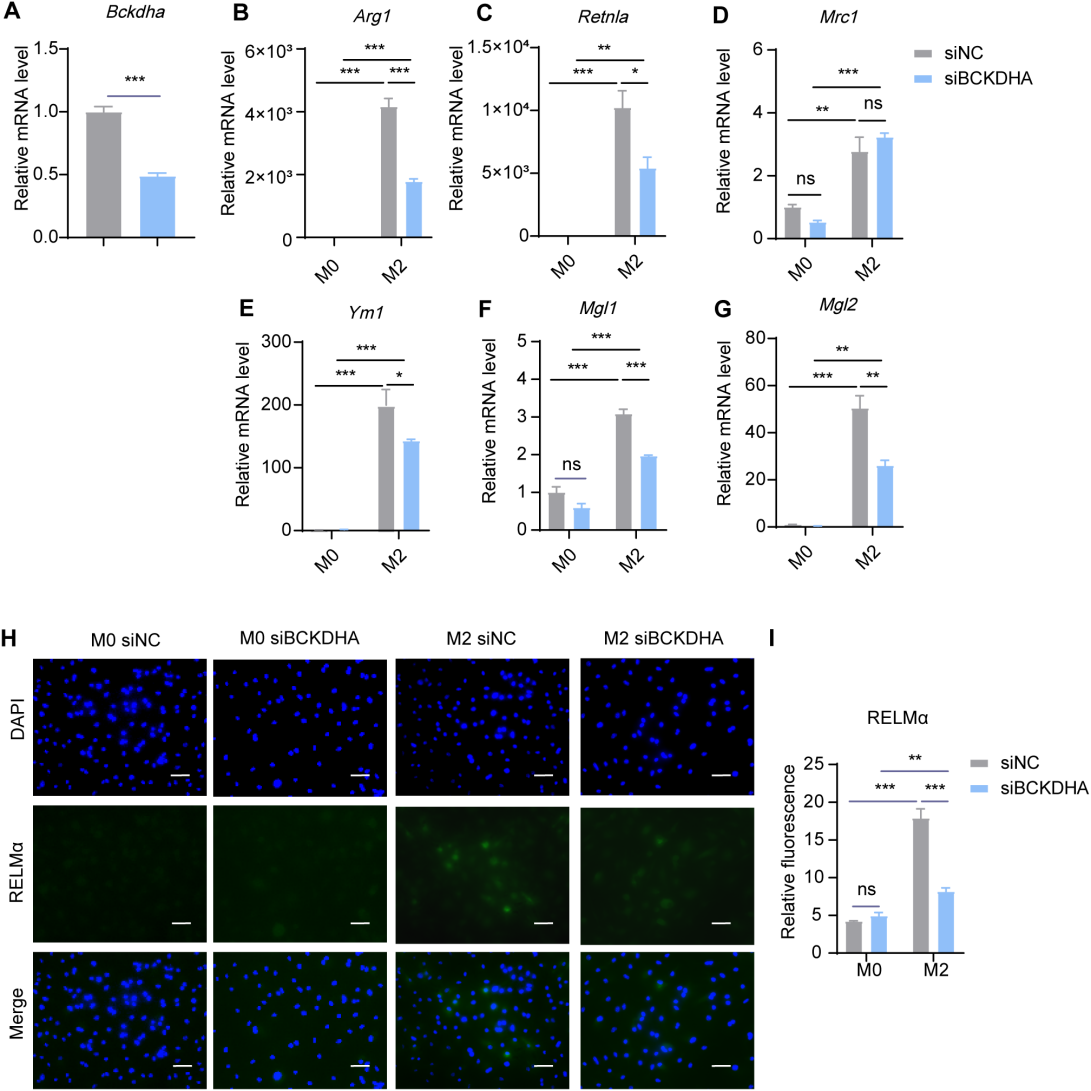
*Bckdha* knockdown inhibits M2 polarization. BMDMs were transfected with siRNA targeting *Bckdha* (siBCKDHA) and scrambled control siRNA (siNC) followed by M2 polarization. The mRNA expression levels of *Bckdha* **(A)**, *Arg1* **(B)**, *Retnla* **(C)**, *Mrc1* **(D)**, *Ym1* **(E)**, *Mgl1* **(F)** and *Mgl2* **(G)** were assessed by RT-qPCR (n = 3 replicates). **(H)** BMDMs were treated with IL-4 (20 ng/mL) or not for 24 hr and subjected to immunofluorescence using anti-RELMα antibody (green) and DAPI (blue). Scale bar, 20 μm. **(I)** Quantification of relative fluorescent signal for RELMα in BMDMs from siNC and siBCKDHA groups (n = 5 replicates). Data are shown as mean ± SEM. Statistics were performed using two way ANOVA followed by Tukey multiple comparisons. *P < 0.05; **P < 0.01; ***P < 0.001; ns, not significant. *Arg1*, Arginase 1; *Retnla*, Resistin like alpha; *Mrc1*, Mannose receptor, C type 1; *Ym1*, Chitinase 3 like 1; *Mgl1*, Macrophage galactose N-acetyl-galactosamine specific lectin 1; *Mgl2*, Macrophage galactose N-acetyl-galactosamine specific lectin 2.

BCKAs undergo oxidative decarboxylation mediated by the BCKDH complex, comprising catalytic subunits E1α, E1β, E2 and E3 encoded by the BCKDHA, BCKDHB, BCKDE2 and BCKDE3 genes, respectively. We then explored the role of BCKA decarboxylation in M2 macrophage polarization. Using siRNA, we knocked down *Bckdha* in BMDMs and induced M2 macrophage polarization. The results revealed that *Bckdha* knockdown reduced the expression levels of M2 marker genes, including *Arg1*, *Retnla*, *Ym1*, *Mgl1* and *Mgl2*, while the expression level of *Mrc1* remained unchanged (**Figures 8A–G**). Additionally, the protein level of RELMα was decreased in M2 macrophages following *Bckdha* knockdown (**Figures 8H, I**). These results suggested that inhibiting BCKDH-mediated BCKA decarboxylation reduces BMDM M2 polarization.

## 4 Discussions

Macrophages modulate their metabolic status to adapt to different activation status, including M1 and M2 activation status. In our study, we found that BCAA catabolism promoted polarization toward M2 macrophages. Metabolomic analysis revealed that the abundance of BCAAs is reduced in M2 polarized macrophages. BCAAs promoted M2 macrophage polarization both *in vitro* and *in vivo*. Furthermore, knockdown of key genes involved in BCAA catabolism was shown to suppress polarization toward M2 macrophages (**Supplementary Figure 9**). Our study therefore provided evidence that BCAA metabolism has a role in M2 macrophage polarization and suggested that BCAA treatment may provide a therapeutic strategy for stimulating M2 macrophages-mediated tissue repair or remodeling.

The question remains whether BCAA catabolism specifically modulate M2 macrophage polarization. Indeed, previous studies have indicated that BCAA metabolism modulates M1 polarization. For example, BCAAs has been shown to promote M1 polarization and enhanced mTORC1-HIFα signaling in M1 macrophages (17). Furthermore, the BCAT1 inhibitor ERG240 has been reported to reduce LPS induced M1 polarization (15). Investigating the impact of BCAA catabolism on M1 polarization would be a valuable direction for future research. Additionally, this study focused on IL-4-induced M2 polarization, as it is the most extensively studied type of polarized M2 macrophages. Investigating the effects of BCAA on other M2 macrophage subtypes stimulated by IL-10, IL-4+IL-10, TGFβ, and other factors would be valuable and will be addressed in future research.

In the study, we detected the mRNA expression level of BCAA metabolism genes such as *Bcat1*, *Bcat2*, *Bckdha* and *Slc25a44*, and the protein level of key enzyme BCKDHA to investigate whether BCAA metabolism pathway is upregulated in M2 macrophages. However, the mRNA level of *Bcat2*, *Bckdha* and *Slc25a44* all decreased. It would be informative to assess the metabolic flux of BCAAs using isotope-assisted metabolomics to accurately monitor the catabolic flux of BCAAs in M0 and M2 macrophages (26).

We observed that BCAA supplementation and genetic ablation of key enzymes or the transporter resulted in differential regulation of M2 markers. For instance, BCAA supplementation led to increased expression of *Arg1* and *Reltna*, but decreased *Ym1* expression *in vitro*. In addition, BCAAs were shown to increased *Ym1* expression in PECs in the *in vivo* model of chitin administration, which differs from their *in vitro* effects. This discrepancy may due to the fact that chitin administration can lead to accumulations of eosinophils, basophils, neutrophils (27), as well as increased levels of chemokines and lipid mediators (20, 28, 29). While BCAAs have been shown to suppress *Ym1* expression *in vitro*, they may, in contrast, promote immune cell recruitment or enhance the production of chemokines or lipid mediators *in vivo*, which could lead to increased *Ym1* expression. Further studies are needed to explore whether BCAAs contribute to eosinophil activation.

Metabolism is intricately linked to immune homeostasis, as evidenced by the polarization of macrophages into different states. Our data showed that the inhibition of key enzymes in BCAA catabolism dampens M2 macrophage polarization, suggesting that BCAA catabolism contributes to this process. Interestingly, glioblastoma cells were shown to excrete large amounts of branched-chain ketoacids (BCKAs), which can inhibit the phagocytic activity associated with the typical M1 macrophage phenotype (14). Furthermore, the leucine metabolite ketoisocaproate (KIC) and the isoleucine metabolite α-keto-β-methylvalerate (KMV) have been shown to increase the expression level of *Arg1* in macrophages (30). The question of how BCAA catabolism promotes M2 macrophages polarization remains. We speculate that BCAA may fuel the TCA cycle, which is suggested to be critical for M2 macrophage polarization (25, 31–33). This idea is supported by the fact that BCAA catabolic metabolites enter the TCA cycle as acetyl coenzyme A (acetyl-CoA) or succinyl coenzyme A (succinyl-CoA). It is well established that mitochondrial OXPHOS is characteristic and necessary for ATP production and biosynthesis in M2 macrophages (25, 32, 33). Inhibition of OXPHOS by reducing substrates or targeting the mitochondrial complex had been shown to downregulate M2-related genes (*Arg1*, *Mrc1*) and the surface marker CD206 (32, 34). Fatty acid oxidation fuels OXPHOS with acetyl-CoA, thus providing a crucial energy source for M2 polarization (25). In our study, BCAA supplementation was found to promote M2 polarization and enhance OXPHOS during this process, revealing a possibility that BCAA may promote macrophage M2 polarization through enhancing OXPHOS. Previous study has shown that BCAA increases oxygen consumption rate (OCR) in brown adipocytes, which actively utilizes BCAA in the mitochondria for oxidation and thermogenesis upon cold exposure (10). Additionally, a mitochondrial-targeted 2C-type Ser/Thr protein phosphatase (PPM1K), which promotes BCAA catabolism, was suggested to sustain glycolysis and oxidative phosphorylation in hematopoietic stem cells (35).

In addition to its role as an energy source in macrophage M2 polarization, acetyl-CoA serves as a critical substrate for histone acetylation, promoting an open chromatin structure that facilitates the transcription of anti-inflammatory and tissue repair genes characteristic of M2 macrophages (36–38). For instance, histone deacetylase 3 (HDAC3) removes acetylation marks from regulatory regions of M2 genes, thereby repressing M2 polarization and promoting M1 macrophage activation (39, 40). Moreover, acetyl-CoA synthesis by ACLY has been shown to be crucial for histone acetylation and the transcriptional induction of a subset of M2 genes (41). Based on our study, which showed that inhibition of BCAA catabolism suppressed M2 macrophage polarization, and the abundance of acetyl-CoA increased in M2 macrophages, we hypothesize that BCAA catabolism might also modulate transcription regulation of M2 genes via their metabolite, acetyl-CoA. Further investigations are needed to elucidate the specific role of BCAAs in producing metabolic products, particularly acetyl-CoA, during M2 macrophage polarization. An earlier study has shown that the inhibition of p300, a histoneacetyltransferase (HAT), suppresses most M2 marker genes, while promoting *Ym1* expression (41). This indicates that histone acetylation may suppress *Ym1* expression in M2 macrophages. Notably, BCAA catabolic metabolites enter the TCA cycle as acetyl-CoA (9), which serves as a critical substrate for histone acetylation, enhancing the transcription of the M2 marker genes (41). Based on this, we speculate that BCAAs suppress *Ym1* expression, potentially through mechanisms related to acetyl-CoA production and histone acetylation, which are still unclear and warrant further investigation.

M2 macrophages are important players in tissue repair following injury, making them potential target for treating tissue repair-related diseases. For instance, during acute liver injury (ALI), which is caused by endotoxins, certain drugs and their metabolites, or other factors. M2 macrophages can mitigate liver damage through their anti-inflammatory properties and ability to promote tissue repair (42). Existing research has found that regulating macrophage polarization can modulate ALIs. For example, activation of cannabinoid receptor 2 (CB2) has been shown to attenuate d-Galactosamine (GalN)/lipopolysaccharide (LPS)-induced acute liver failure by inducing an M1 to M2 shift in macrophages (43). Similarly, inhibition of p38α has been found to induce a shift from M1 to M2 macrophages polarization, thereby alleviating liver injury (44). Our observation that BCAA promotes M2 macrophage polarization both *in vitro* and *in vivo* indicates that BCAA supplementation may in part enhance liver repair by promoting M2 macrophage polarization. Indeed, supplementation with BCAA has been shown to activate Kupffer cells and attenuated ischemia-reperfusion-induced hepatic leukocyte adhesion and hepatic microcirculation disturbances in rats (45). Given that BCAA supplementation has been reported to promote M1 macrophage polarization, it is still premature to conclude that BCAAs enhance M2 immunity in pathological settings that require M2 polarization. Further studies in additional pathophysiological models are needed to better understand the effects of BCAAs on M2 polarization in these contexts and to explore the potential of BCAA supplementation as a therapeutic strategy for tissue repair-related diseases.

## Data Availability

The raw data supporting the conclusions of this article will be made available by the authors, without undue reservation.

## References

[1] Lazarov T Juarez-Carreño S Cox N Geissmann F. Physiology and diseases of tissue-resident macrophages. Nature. (2023) 618:698–707. doi: 10.1038/s41586-023-06002-x 37344646 PMC10649266

[2] Shapouri-Moghaddam A Mohammadian S Vazini H Taghadosi M Esmaeili SA Mardani F. Macrophage plasticity, polarization, and function in health and disease. J Cell Physiol. (2018) 233:6425–40. doi: 10.1002/jcp.26429 PMC13482560 29319160

[3] Sica A Mantovani A. Macrophage plasticity and polarization: *in vivo* veritas. J Clin Invest. (2012) 122:787–95. doi: 10.1172/jci59643 PMC3287223 22378047

[4] Kreider T Anthony RM Urban JF Jr. Gause WC. Alternatively activated macrophages in helminth infections. Curr Opin Immunol. (2007) 19:448–53. doi: 10.1016/j.coi.2007.07.002 PMC2000338 17702561

[5] Xie Y Zhou X Zhang J Yu H Song Z. Immunomodulatory responses of differentially polarized macrophages to fungal infections. Int Immunopharmacol. (2022) 111:109089. doi: 10.1016/j.intimp.2022.109089 35964406

[6] Wculek SK Dunphy G Heras-Murillo I Mastrangelo A Sancho D. Metabolism of tissue macrophages in homeostasis and pathology. Cell Mol Immunol. (2022) 19:384–408. doi: 10.1038/s41423-021-00791-9 34876704 PMC8891297

[7] Rath M Müller I Kropf P Closs EI Munder M. Metabolism via arginase or nitric oxide synthase: two competing arginine pathways in macrophages. Front Immunol. (2014) 5:532. doi: 10.3389/fimmu.2014.00532 25386178 PMC4209874

[8] Ryan DG O'Neill LAJ. Krebs cycle reborn in macrophage immunometabolism. Annu Rev Immunol. (2020) 38:289–313. doi: 10.1146/annurev-immunol-081619-104850 31986069

[9] Neinast M Murashige D Arany Z. Branched chain amino acids. Annu Rev Physiol. (2019) 81:139–64. doi: 10.1146/annurev-physiol-020518-114455 PMC6536377 30485760

[10] Yoneshiro T Wang Q Tajima K Matsushita M Maki H Igarashi K. Bcaa catabolism in brown fat controls energy homeostasis through slc25a44. Nature. (2019) 572:614–9. doi: 10.1038/s41586-019-1503-x PMC6715529 31435015

[11] Delgado-Martin M Zhang Q Kazak L. Bat-tling oxidative stress through bcaa catabolism. Life Metab. (2024) 3:loae028. doi: 10.1093/lifemeta/loae028 PMC11748966 39872145

[12] Nong X Zhang C Wang J Ding P Ji G Wu T. The mechanism of branched-chain amino acid transferases in different diseases: research progress and future prospects. Front Oncol. (2022) 12:988290. doi: 10.3389/fonc.2022.988290 36119495 PMC9478667

[13] White PJ McGarrah RW Herman MA Bain JR Shah SH Newgard CB. Insulin action, type 2 diabetes, and branched-chain amino acids: A two-way street. Mol Metab. (2021) 52:101261. doi: 10.1016/j.molmet.2021.101261 34044180 PMC8513145

[14] Silva LS Poschet G Nonnenmacher Y Becker HM Sapcariu S Gaupel AC. Branched-chain ketoacids secreted by glioblastoma cells via mct1 modulate macrophage phenotype. EMBO Rep. (2017) 18:2172–85. doi: 10.15252/embr.201744154 PMC5709768 29066459

[15] Papathanassiu AE Ko JH Imprialou M Bagnati M Srivastava PK Vu HA. Bcat1 controls metabolic reprogramming in activated human macrophages and is associated with inflammatory diseases. Nat Commun. (2017) 8:16040. doi: 10.1038/ncomms16040 28699638 PMC5510229

[16] Zhenyukh O Civantos E Ruiz-Ortega M Sánchez MS Vázquez C Peiró C. High concentration of branched-chain amino acids promotes oxidative stress, inflammation and migration of human peripheral blood mononuclear cells via mtorc1 activation. Free Radic Biol Med. (2017) 104:165–77. doi: 10.1016/j.freeradbiomed.2017.01.009 28089725

[17] Dong Y Zhang X Miao R Cao W Wei H Jiang W. Branched-chain amino acids promotes the repair of exercise-induced muscle damage via enhancing macrophage polarization. Front Physiol. (2022) 13:1037090. doi: 10.3389/fphys.2022.1037090 36561213 PMC9763461

[18] Ying W Cheruku PS Bazer FW Safe SH Zhou B. Investigation of macrophage polarization using bone marrow derived macrophages. J Vis Exp. (2013) 76:196–202. doi: 10.3791/50323 PMC3728835 23851980

[19] Ji L Zhao X Zhang B Kang L Song W Zhao B. Slc6a8-mediated creatine uptake and accumulation reprogram macrophage polarization via regulating cytokine responses. Immunity. (2019) 51:272–84.e7. doi: 10.1016/j.immuni.2019.06.007 31399282

[20] Satoh T Takeuchi O Vandenbon A Yasuda K Tanaka Y Kumagai Y. The jmjd3-irf4 axis regulates M2 macrophage polarization and host responses against helminth infection. Nat Immunol. (2010) 11:936–44. doi: 10.1038/ni.1920 20729857

[21] Galsgaard KD Jepsen SL Kjeldsen SAS Pedersen J Wewer Albrechtsen NJ Holst JJ. Alanine, Arginine, Cysteine, and Proline, but Not Glutamine, Are Substrates for, and Acute Mediators of, the Liver-α-Cell Axis in Female Mice. Am J Physiol Endocrinol Metab. (2020) 318:E920–e9. doi: 10.1152/ajpendo.00459.2019 32255678

[22] Li J Dong Y Zhou T Tian H Huang X Zhang YQ. Long-chain acyl-coa synthetase regulates systemic lipid homeostasis via glycosylation-dependent lipoprotein production. Life Metab. (2024) 3:2097–555. doi: 10.1093/lifemeta/loae004 PMC11749247 39872215

[23] Bijlsma S Bobeldijk I Verheij ER Ramaker R Kochhar S Macdonald IA. Large-scale human metabolomics studies: A strategy for data (Pre-) processing and validation. Anal Chem. (2006) 78:567–74. doi: 10.1021/ac051495j 16408941

[24] Rampler E Abiead YE Schoeny H Rusz M Hildebrand F Fitz V. Recurrent topics in mass spectrometry-based metabolomics and lipidomics-standardization, coverage, and throughput. Anal Chem. (2021) 93:519–45. doi: 10.1021/acs.analchem.0c04698 PMC7807424 33249827

[25] Huang SC Everts B Ivanova Y O'Sullivan D Nascimento M Smith AM. Cell-intrinsic lysosomal lipolysis is essential for alternative activation of macrophages. Nat Immunol. (2014) 15:846–55. doi: 10.1038/ni.2956 PMC4139419 25086775

[26] Jang C Chen L Rabinowitz JD. Metabolomics and isotope tracing. Cell. (2018) 173:822–37. doi: 10.1016/j.cell.2018.03.055 PMC6034115 29727671

[27] Reese TA Liang HE Tager AM Luster AD Van Rooijen N Voehringer D. Chitin induces accumulation in tissue of innate immune cells associated with allergy. Nature. (2007) 447:92–6. doi: 10.1038/nature05746 PMC2527589 17450126

[28] Brodaczewska K Donskow-Łysoniewska K Doligalska M. Chitin, a key factor in immune regulation: lesson from infection with fungi and chitin bearing parasites. Acta Parasitol. (2015) 60:337–44. doi: 10.1515/ap-2015-0047 26204004

[29] Elieh Ali Komi D Sharma L Dela Cruz CS. Chitin and its effects on inflammatory and immune responses. Clin Rev Allergy Immunol. (2018) 54:213–23. doi: 10.1007/s12016-017-8600-0 PMC5680136 28251581

[30] Cai Z Li W Brenner M Bahiraii S Heiss EH Weckwerth W. Branched-chain ketoacids derived from cancer cells modulate macrophage polarization and metabolic reprogramming. Front Immunol. (2022) 13:966158. doi: 10.3389/fimmu.2022.966158 36311795 PMC9606345

[31] Kieler M Hofmann M Schabbauer G. More than just protein building blocks: how amino acids and related metabolic pathways fuel macrophage polarization. FEBS J. (2021) 288:3694–714. doi: 10.1111/febs.15715 PMC8359336 33460504

[32] Vats D Mukundan L Odegaard JI Zhang L Smith KL Morel CR. Oxidative metabolism and pgc-1beta attenuate macrophage-mediated inflammation. Cell Metab. (2006) 4:13–24. doi: 10.1016/j.cmet.2006.05.011 16814729 PMC1904486

[33] Loftus RM Finlay DK. Immunometabolism: cellular metabolism turns immune regulator. J Biol Chem. (2016) 291:1–10. doi: 10.1074/jbc.R115.693903 26534957 PMC4697146

[34] Van den Bossche J Baardman J Otto NA van der Velden S Neele AE van den Berg SM. Mitochondrial dysfunction prevents repolarization of inflammatory macrophages. Cell Rep. (2016) 17:684–96. doi: 10.1016/j.celrep.2016.09.008 27732846

[35] Liu X Zhang F Zhang Y Li X Chen C Zhou M. Ppm1k regulates hematopoiesis and leukemogenesis through cdc20-mediated ubiquitination of meis1 and P21. Cell Rep. (2018) 23:1461–75. doi: 10.1016/j.celrep.2018.03.140 29719258

[36] Aaron N Zahr T He Y Yu L Mayfield B Pajvani UB. Acetylation of pparγ in macrophages promotes visceral fat degeneration in obesity. Life Metab. (2022) 1:258–69. doi: 10.1093/lifemeta/loac032 PMC10198133 37213714

[37] Verberk SG de Goede KE Van den Bossche J. Metabolic-epigenetic crosstalk in macrophage activation: an updated view. Epigenomics. (2019) 11:719–21. doi: 10.2217/epi-2019-0073 31150278

[38] Li N Horng T. A metabolic conspiracy drives anti-tumorigenic macrophages. Life Metab. (2023) 2:2755–0230. doi: 10.1093/lifemeta/load009 PMC11748994 39872733

[39] Hoeksema MA Gijbels MJ Van den Bossche J van der Velden S Sijm A Neele AE. Targeting macrophage histone deacetylase 3 stabilizes atherosclerotic lesions. EMBO Mol Med. (2014) 6:1124–32. doi: 10.15252/emmm.201404170 PMC4197860 25007801

[40] Mullican SE Gaddis CA Alenghat T Nair MG Giacomin PR Everett LJ. Histone deacetylase 3 is an epigenomic brake in macrophage alternative activation. Genes Dev. (2011) 25:2480–8. doi: 10.1101/gad.175950.111 PMC3243058 22156208

[41] Covarrubias AJ Aksoylar HI Yu J Snyder NW Worth AJ Iyer SS. Akt-mtorc1 signaling regulates acly to integrate metabolic input to control of macrophage activation. Elife. (2016) 5:e11612. doi: 10.7554/eLife.11612 26894960 PMC4769166

[42] Wang C Ma C Gong L Guo Y Fu K Zhang Y. Macrophage polarization and its role in liver disease. Front Immunol. (2021) 12:803037. doi: 10.3389/fimmu.2021.803037 34970275 PMC8712501

[43] Tomar S Zumbrun EE Nagarkatti M Nagarkatti PS. Protective role of cannabinoid receptor 2 activation in galactosamine/lipopolysaccharide-induced acute liver failure through regulation of macrophage polarization and micrornas. J Pharmacol Exp Ther. (2015) 353:369–79. doi: 10.1124/jpet.114.220368 PMC4407720 25749929

[44] Liu J Zhang S Cao H Wang H Sun C Liu S. Deficiency of P38α in macrophage ameliorates D-galactosamine/tnf-α-induced acute liver injury in mice. FEBS J. (2017) 284:4200–15. doi: 10.1111/febs.14294 29052963

[45] Kitagawa T Yokoyama Y Kokuryo T Nagino M. Protective effects of branched-chain amino acids on hepatic ischemia-reperfusion-induced liver injury in rats: A direct attenuation of kupffer cell activation. Am J Physiol Gastrointest Liver Physiol. (2013) 304:G346–55. doi: 10.1152/ajpgi.00391.2012 23275614

