# Supplementary material for: Branched-chain amino acid catabolism promotes M2 macrophage polarization
Source: Front Immunol. 2024 Nov 8;15:1469163. doi: 10.3389/fimmu.2024.1469163 (PMC11582057; doi:10.3389/fimmu.2024.1469163)
Supplement: Supplementary file 1 [file DataSheet1.docx]

Supplementary Material

# Supplementary Figures and Tables

## Supplementary Figures


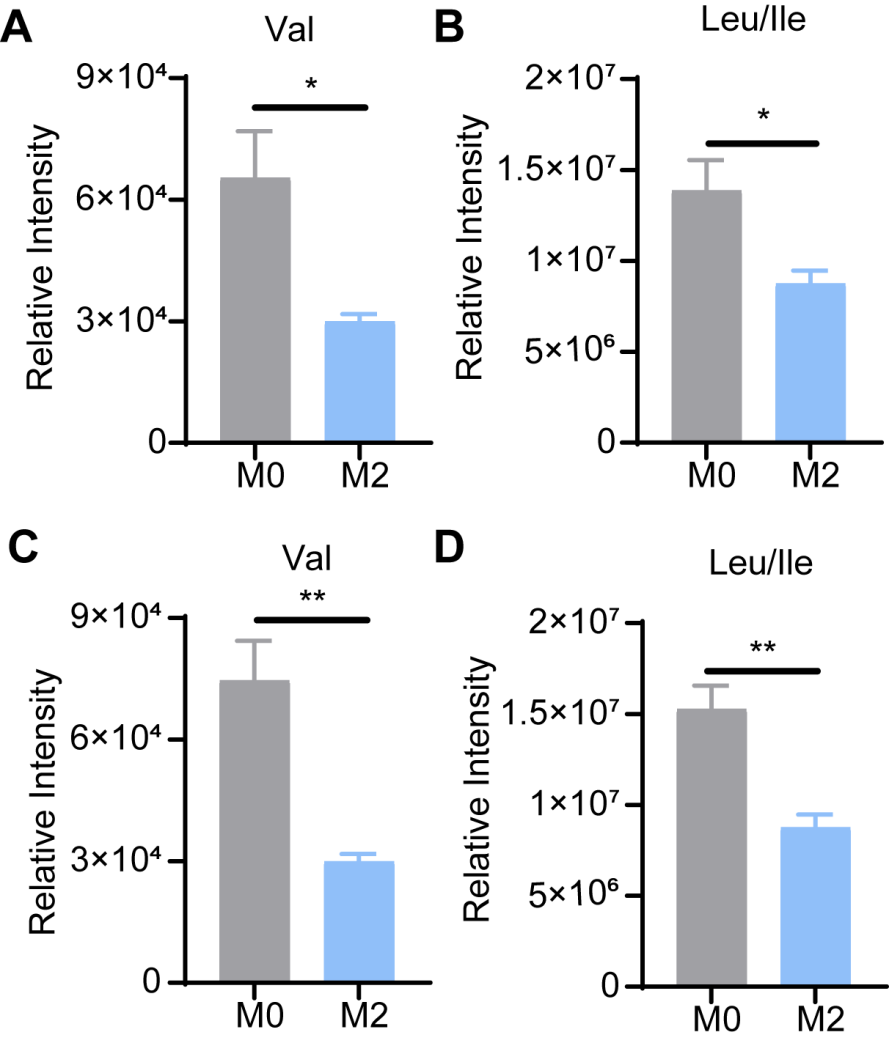


**Supplementary Figure 1. The relative intensity of Val and Leu/Ile with or without M0 outlier. (A-B)** Relative intensity of Val and Leu/Ile with M0 outlier (n = 4 replicates)**. (C-D)** Relative intensity of Val and Leu/Ile without M0 outlier (n = 4 replicates). Data are shown as mean ± SEM. Statistics were performed using two-tailed Student’s *t* test. **P* < 0.05; ***P* < 0.01.


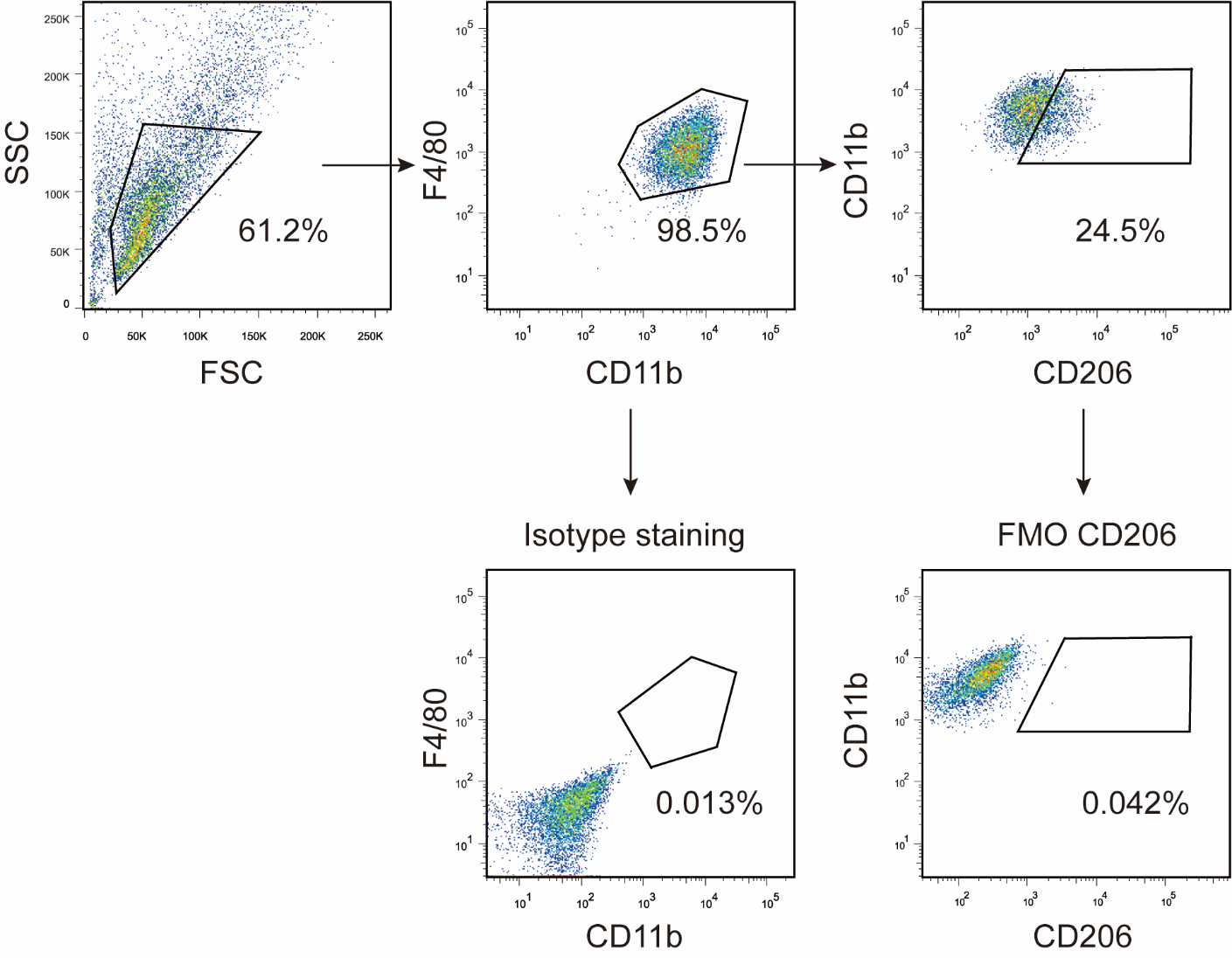


**Supplementary Figure 2**. **Gating strategy, isotype and FMO controls for *in vitro* polarized M2 macrophages.** *In vitro* polarized M2 macrophages were pre-gated on FSC/SSC parameters, and then gated for CD11b^+^F4/80^+^ cells. Isotype controls for CD11b and F4/80 and FMO control for CD206 were presented.


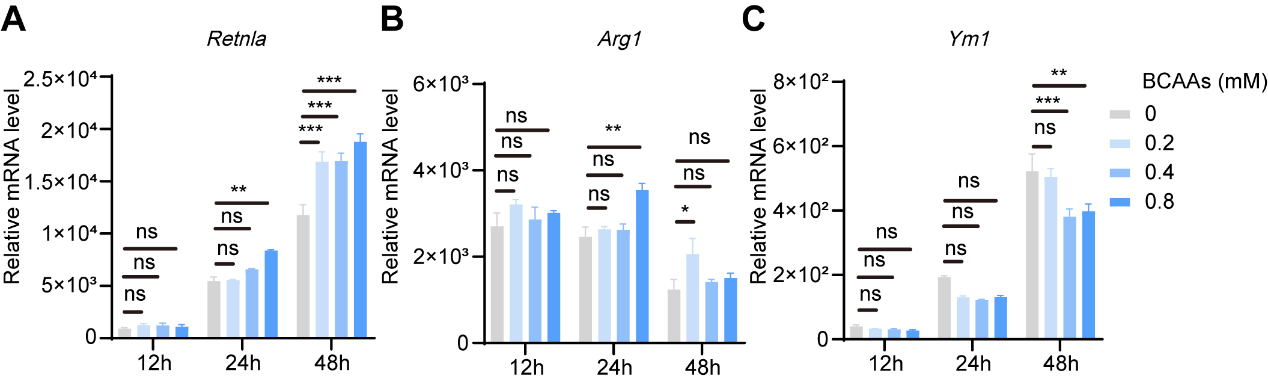


**Supplementary Figure 3. Time and dose-dependent effect of BCAAs on the expression of M2 maker genes**. BMDMs were treated with 0, 0.2, 0.4 and 0.8 mM BCAAs in the presence of IL-4 for the indicated time period. The mRNA expression levels of *Retnla* (A), *Arg1* (B) and *Ym1* (C) were assessed by RT-qPCR and and normalized to the expression levels in M0 macrophages (n = 3 replicates). Statistics were performed using two way ANOVA followed by Tukey multiple comparisons. **P* < 0.01; ***P* < 0.01; ****P* < 0.001; ns, not significant. *Retnla*, Resistin like alpha; *Arg1*, Arginase 1; *Ym1*, Chitinase 3 like 1.


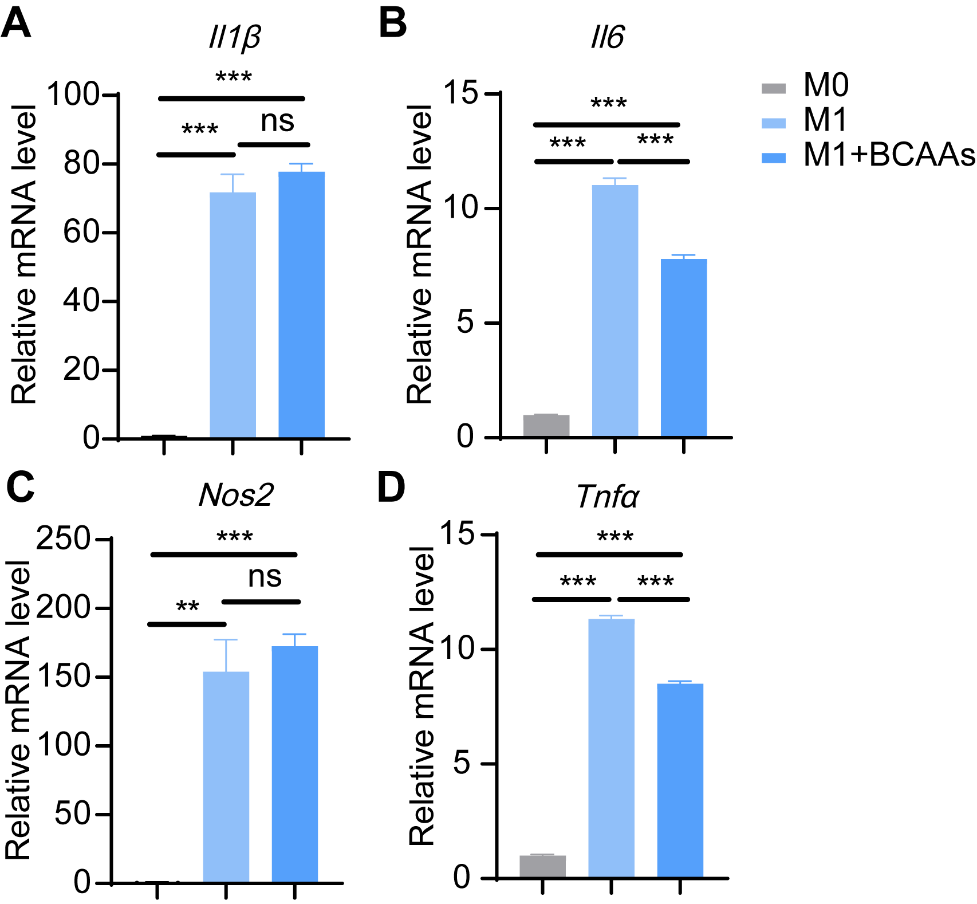


**Supplementary Figure 4.** **The effect of BCAAs on M1 macrophage polarization.** BMDMs were cultured under M0 condition or under M1 condition (100 ng/mL LPS and 50 ng/mL IFNγ) with 0.8 mM BCAAs or vehicle for 24 hr. mRNA expression levels of *Il1β* (A), *Il6* (B), *Nos2* (C), *Tnfa* (D) were assessed by RT-qPCR (n = 3 replicates). Data are shown as mean ± SEM. Statistics were performed using two-tailed Student’s *t* test. ***P* < 0.01; ****P* < 0.001; ns, not significant. *Il1β*, interleukin 1 beta; *Il6,* interleukin 6; *Nos2,* nitric oxide synthase 2; *Tnfa,* tumor necrosis factor *a.*


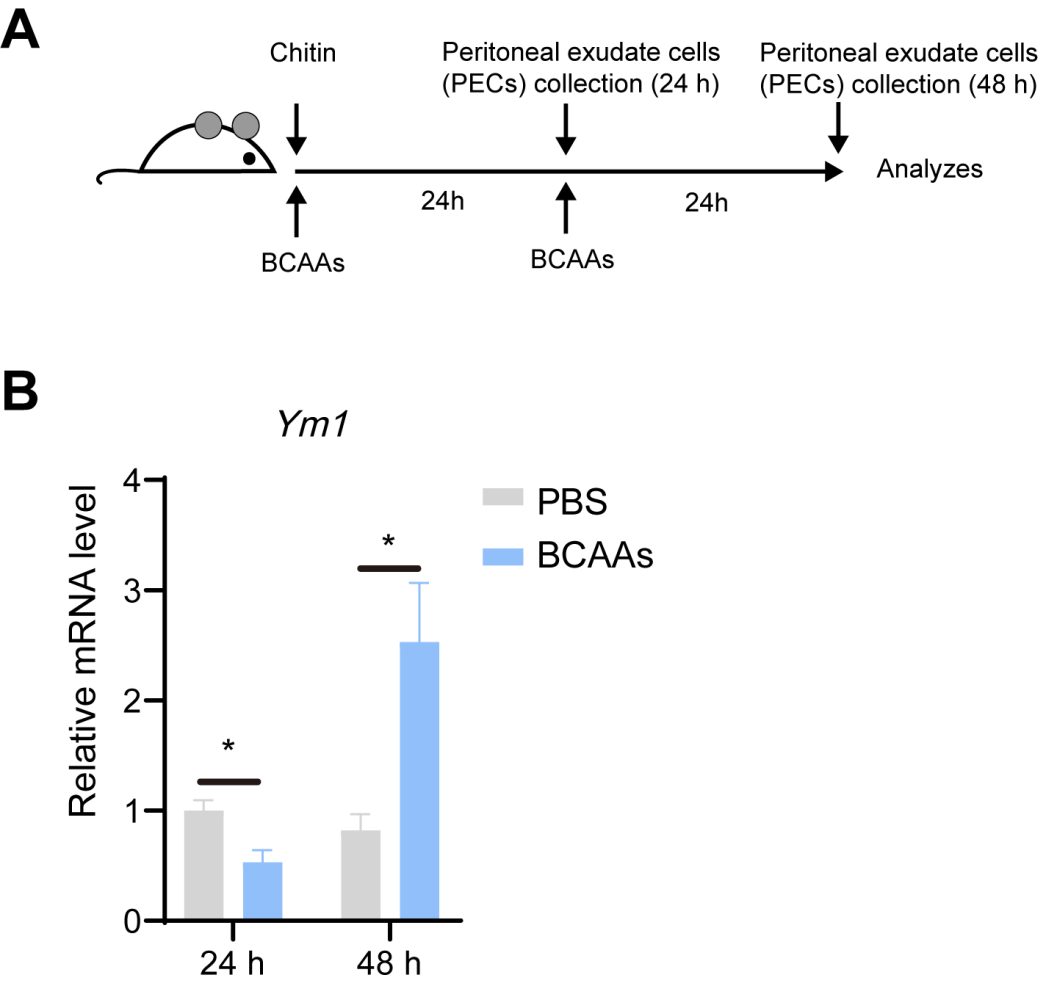


**Supplementary Figure 5. The effect of BCAA supplementation on *Ym1* expression in PECs after chitin administration.** (A) Schematic diagram of the experimental protocol in 6-8 week-old female mice. Mice were intraperitoneally administrated with 100 μL chitin and 90 μmol were administrated at 0 and 24 hr after chitin administration. PECs were collected and analyzed at 24 and 48 hr post chitin administration. **(B)** mRNA expression levels of *Ym1* in PECs was determined by quantitative PCR (n = 4 independent biological replicates). Data are shown as mean ± SEM. Statistics were performed using two-tailed Student’s *t* test. **P* < 0.05. *Ym1*, Chitinase 3 like 1.


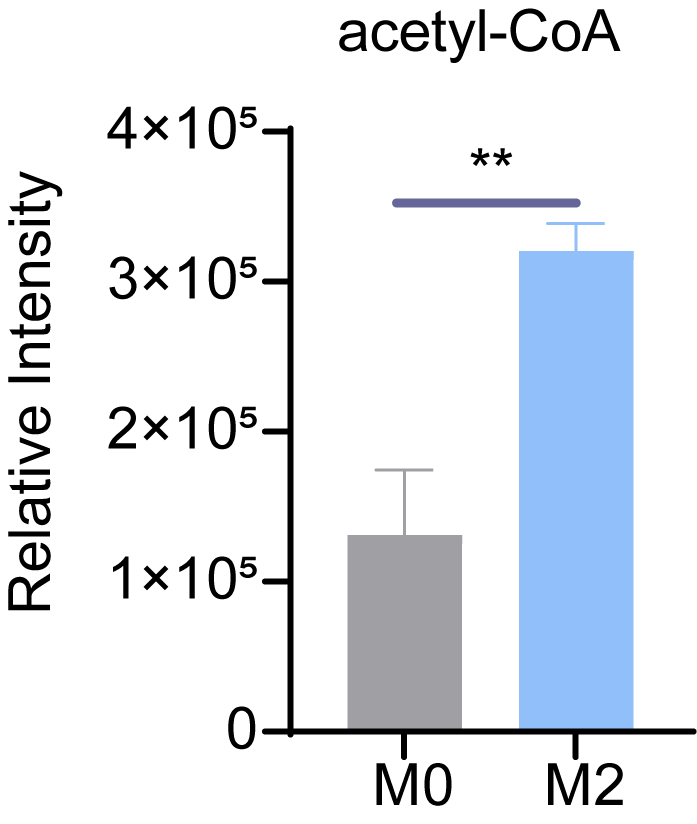


**Supplementary Figure 6.** The relative intensity of acetyl-CoA in M0 and M2 BMDMs as determined by metabolomic analysis (n = 4 replicates). Data are shown as mean ± SEM. Statistics were performed using two-tailed Student’s *t* test. ***P* < 0.01.

**
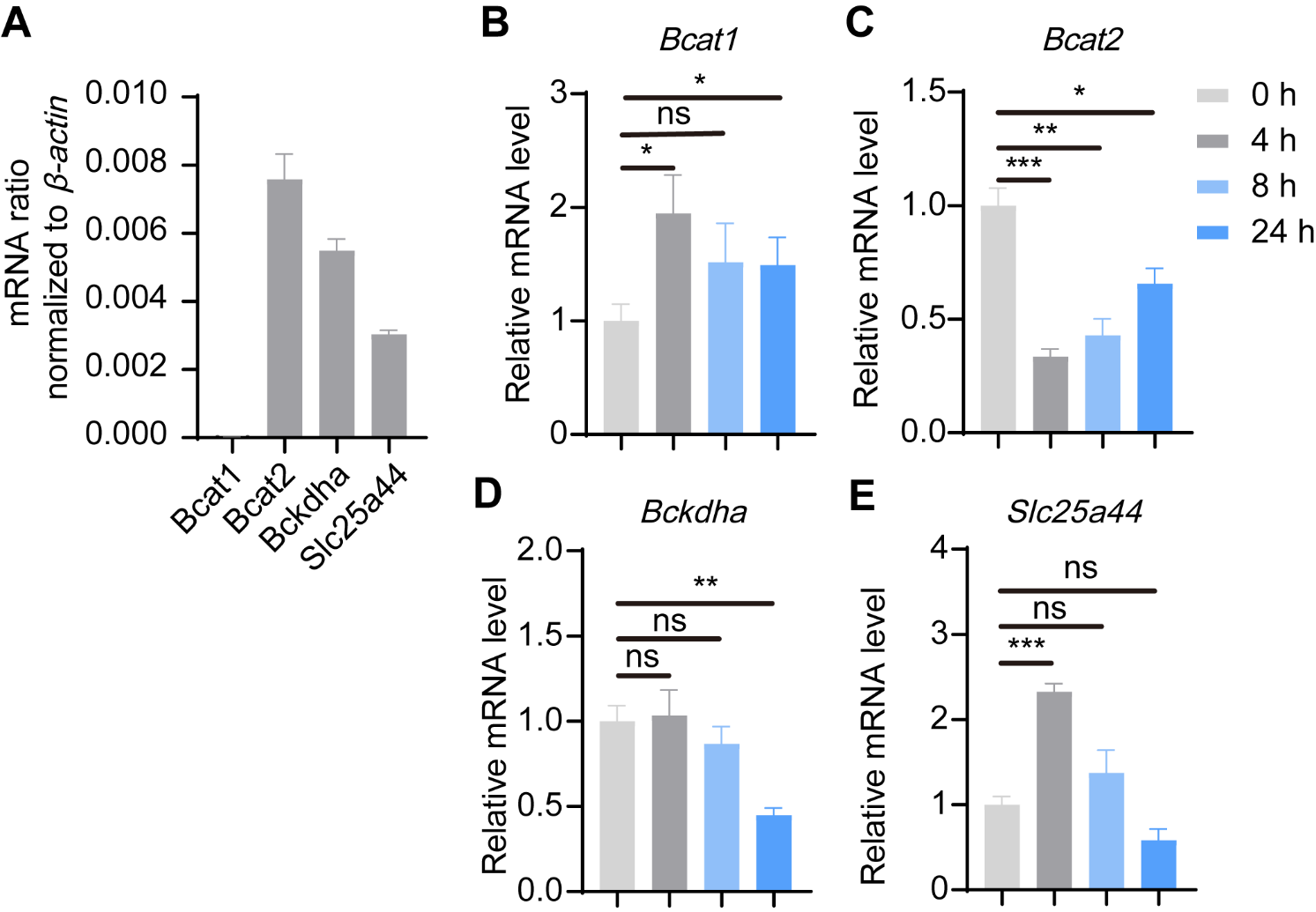
Supplementary Figure 7.** **The expression level of BCAA metabolism related genes after IL-4 stimulation.** (A) mRNA expression levels of *Bcat1*, *Bcat2*, *Bckdha* and *Slc25a44* in M0 BMDM compared to *β-actin*. BMDMs were treated with 20 ng/mL IL-4 stimulation and the mRNA expression levels of *Bcat1* (B), *Bcat2* (C), *Bckdha* (D), *Slc25a44* (E) were assessed by RT-qPCR at indicated time points (n = 4 replicates). Data were shown as mean ± SEM. Statistics were performed using two-tailed Student’s *t* test. **P* < 0.05; ***P* < 0.01; ****P* < 0.001; ns, not significant. *Bcat1*, branched chain aminotransferase 1, cytosolic; *Bcat2*, branched chain aminotransferase 2, mitochondrial; *Bckdha*, branched chain ketoacid dehydrogenase E1, alpha polypeptide; *Slc25a44*, solute carrier family 25, member 44*,*


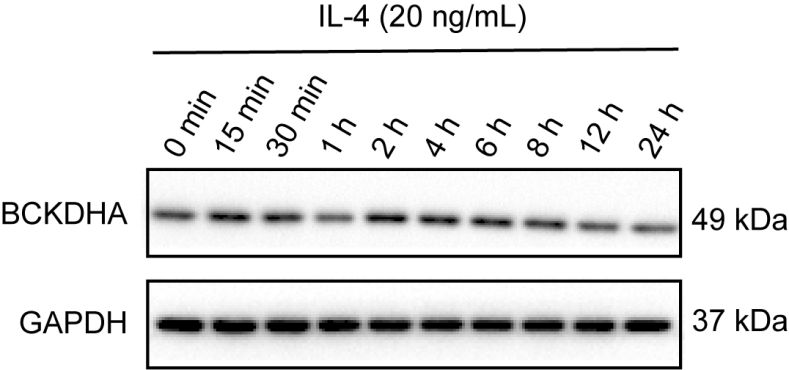


**Supplementary Figure 8.** **Protein level of BCKDHA in BMDMs at different time after IL-4 ( 20 ng/ mL) stimulation.** GAPDH was used as the loading control.


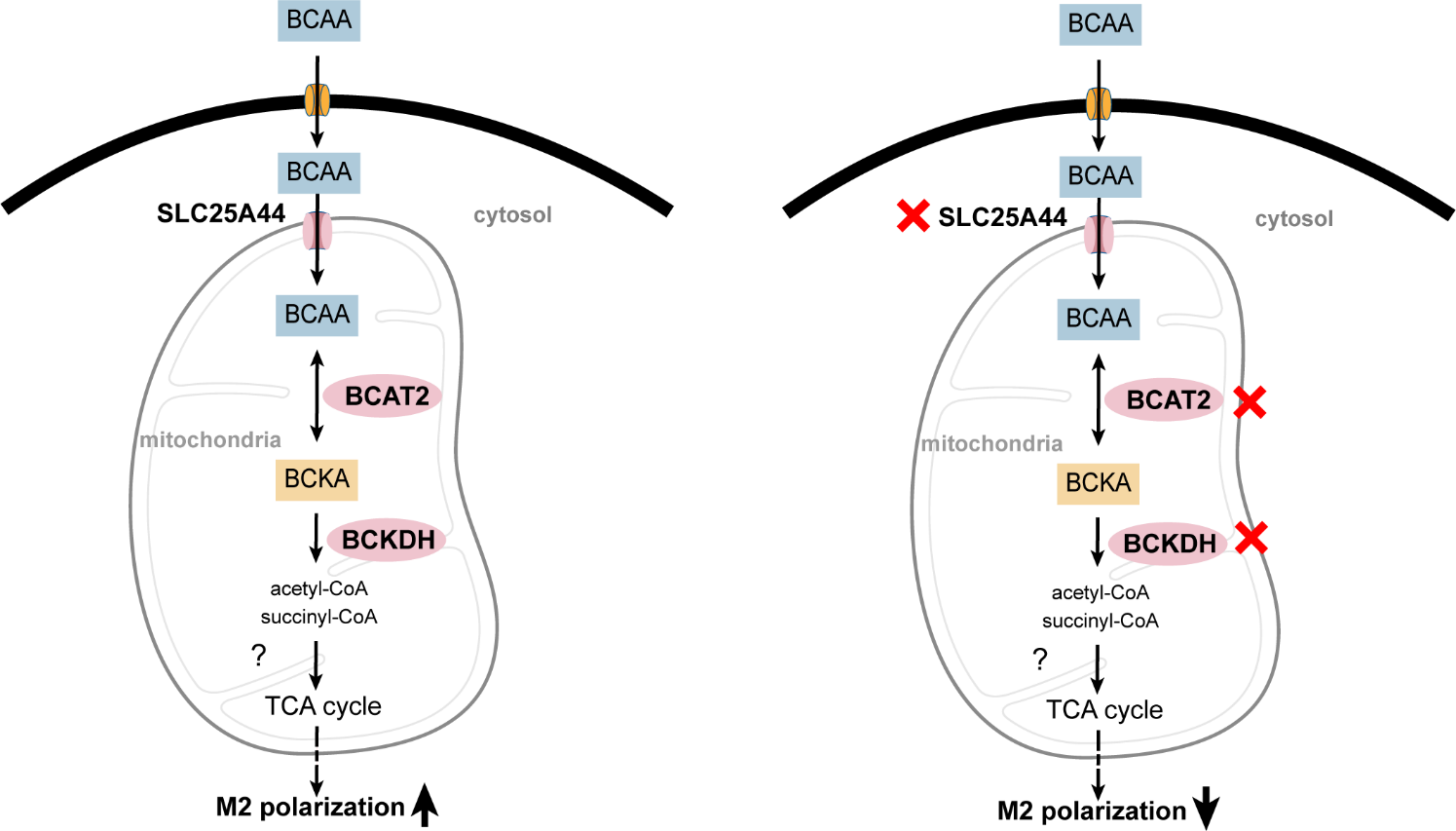


**Supplementary Figure 9.** **Schematic representation of how BCAA metabolism regulate M2 polarization in macrophages.** BCAAs are transported into the mitochondria by SLC25A44, where they are metabolized by BCAT2 and BCKDH before entering the TCA cycle. Blockade of SLC25A44, BCAT2 or BCKDH leads to suppression of M2 macrophage polarization.

**Supplementary Table 1.**

**Primer sequences of real-time quantitative PCR**

| Primer name | Sequence (5' to 3') |
| --- | --- |
| mus_*β-Actin*_For | GGCTGTATTCCCCTCCATCG |
| mus_*β-Actin*_Rev | CCAGTTGGTAACAATGCCATGT |
| mus_*Arg1*_For | CTCCAAGCCAAAGTCCTTAGAG |
| mus_*Arg1*_Rev | AGGAGCTGTCATTAGGGACATC |
| mus_*Retnla*_For | CCAATCCAGCTAACTATCCCTCC |
| mus_*Retnla*_Rev | CCAGTCAACGAGTAAGCACAG |
| mus_*Mrc1*_For | GTCAGAACAGACTGCGTGGA |
| mus_*Mrc1*_Rev | AGGGATCGCCTGTTTTCCAG |
| mus_*Ym1*_For | CCAGCAGAAGCTCTCCAGAAGCA |
| mus_*Ym1*_Rev | TGGTAGGAAGATCCCAGCTGTACG |
| mus_*Mgl1*_For | CAGAATCGCTTAGCCAATGTGG |
| mus_*Mgl1*_Rev | TCCCAGTCCGTGTCCGAAC |
| mus_*Mgl2*_For | TTCAAGAATTGGAGGCCACT |
| mus_*Mgl2*_Rev | CAGACATCGTCATTCCAACG |
| mus_*Bcat1*_For | GAAGTGGCGGAGACTTTTAGG |
| mus_*Bcat1*_Rev | TGGTCAGTAAACGTAGCTCCA |
| mus_*Bcat2*_For | AAAGCATACAAAGGTGGAGACC |
| mus_*Bcat2*_Rev | CGTAGAGGCTCGTTCCGTTG |
| mus_*Bckdha*_For | AGGAGGTGCTGAAGTTCTACC |
| mus_*Bckdha*_Rev | CGCCATAGTTGGTCATGTAGAAG |
| mus_*Slc25a44*_For | TCGCTGCTAACGTACATCCC |
| mus_*Slc25a44*_Rev | AGACAATGTGAGGGCACTCC |
| mus_*Il1β*_For | GCAACTGTTCCTGAACTCAACT |
| mus_*Il1β*_Rev | ATCTTTTGGGGTCCGTCAACT |
| mus_*Il6*_For | TAGTCCTTCCTACCCCAATTTCC |
| mus_*Il6*_Rev | TTGGTCCTTAGCCACTCCTTC |
| mus_*Nos2*_For | GTTCTCAGCCCAACAATACAAGA |
| mus_*Nos2*_Rev | GTGGACGGGTCGATGTCAC |
| mus_*Tnfα*_For | CATCTTCTCAAAATTCGAGTGACAA |
| mus_*Tnfα*_Rev | TGGGAGTAGACAAGGTACAACCC |
|  |  |
